# Cross-Modal Perceptual Organization in Works of Art

**DOI:** 10.1177/2041669520950750

**Published:** 2020-08-27

**Authors:** Liliana Albertazzi, Luisa Canal, Rocco Micciolo, Iacopo Hachen

**Affiliations:** LabExP, Department of Humanities, University of Trento, Italy; Department of Psychology and Cognitive Sciences, University of Trento, Italy; Neuroscience Area, International School for Advanced Studies (SISSA), Trieste, Italy

**Keywords:** color, multisensory/cross-modal processing, music, perceptual organization, shape

## Abstract

This study investigates the existence of cross-modal correspondences between a series of paintings by Kandinsky and a series of selections from Schönberg music. The experiment was conducted in two phases. In the first phase, by means of the Osgood semantic differential, the participants evaluated the perceptual characteristics first of visual stimuli (some pictures of Kandinsky’s paintings, with varying perceptual characteristics and contents) and then of auditory stimuli (musical excerpts taken from the repertoire of Schönberg’s piano works) relative to 11 pairs of adjectives tested on a continuous bipolar scale. In the second phase, participants were required to associate pictures and musical excerpts. The results of the semantic differential test show that certain paintings and musical excerpts were evaluated as semantically more similar, while others were evaluated as semantically more different. The results of the direct association between musical excerpts and paintings showed both attractions and repulsions among the stimuli. The overall results provide significant insights into the relationship between concrete and abstract concepts and into the process of perceptual grouping in cross-modal phenomena.

In recent years, perception studies have experienced a growing interest in cross-modal phenomena. The existence of naturally biased associations among shape, color, sound, taste, touch, and olfactory perception has been consistently shown, though mainly for simple stimuli, with different paradigms and by different methodologies (see e.g., Albertazzi et al., 2012, 2014; Bremner et al., 2012; Gilbert et al., 1996; Hanson-Vaux et al., 2013; Iosifyan et al., 2017; Kemp & Gilbert, 1997; Mankin & Simner, 2017; Marks, 1987a, 1987b; Osterbauer et al., 2005; Sagiv & Ward, 2006; Stein, 2012). The debate on cross-modal phenomena touched on several points, such as the nature, the explanation, and the proper terminology to define them (Deroy & Spence, 2013a, 2013b; Spence, 2011; Spence & Deroy, 2013). Cross-modal correspondences can be defined as naturally biased associations or congruency effects, between attributes or dimensions of stimuli in different sensory modalities (Spector & Maurer, 2008; Spence, 2011; for an overview of the contemporary debates regarding color/shape associations, see Dreksler & Spence, 2019). For example, it has been shown that correspondences exist between auditory pitch and the size and the shape of perceived visual objects (for a replication of a series of studies and further developments, see Parise & Spence, 2009). It has also been shown that the perceived correspondences between dimensions of stimuli in different modalities are described by scales of antonyms (Karwoski & Odbert, 1938; Karwoski et al., 1942; Osgood, 1960; Osgood et al., 1957; for a review, see Oyama et al., 1998). Culture may play a role in the choice of the antonyms: For example, Western music construes pitch according to an up-down spatial relationship (Pratt, 1930; see also Deroy et al., 2018; Lupton, 2018; Salgado-Montejo et al., 2016), while in other languages pitches are construed according to a size scale (small and large, as in Bali and Java), or to an aging scale (young and old, as in the Amazonian basin; Seeger, 1987; see also Albertazzi, Koenderink, et al., 2015). The most interesting novelty in the field of cross-modality, however, has been the shift of attention from the associations verified between simple stimuli of different modalities to processes of perceptual organization (Bayne & Spence, 2014; Sanabria et al., 2005; Spence & Chen, 2012; Spence et al., 2007; Stein & Meredith, 1993). Over the years, a series of stimuli of increasing complexity have been tested providing evidence of the presence of cross-modal *Gestalten* organizing multisensory grouping. For example, cross-modal correspondences have been verified between abstract concepts and color mapping (Albertazzi et al., 2013), between conceptual literary meanings and classical music (Albertazzi, Canal, et al., 2016), between contents of contemporary expressionist painting and Spanish flamenco music (Albertazzi, Canal, & Micciolo, 2015), and between contents of abstract paintings and tactile perceptions (Albertazzi, Bacci, et al., 2016). The last four studies do not make use of methodologies such as reaction times or implicit association tests. They focus on the phenomenological and subjective perceptual experience in first person account, without recurring to sensory-to-sensory or top-down inferential processes. These studies pertain to the Gestalt tradition and explain cross-modal phenomena in terms of grouping (Marks, 1989; Metzger, 1934; Spence, 2015; Watanabe & Shimojo, 2001; regarding the possible involvement of emotion in associations, see Fechner, 1876; Ortlieb, Kügel, & Carbon 2020; Spence, 2019).

For Gestaltists, concepts are neither sets of attributes defining classes of objects (such as cat, bicycle, cathedral; Kass et al., 2015) nor disjunctive concepts, but perceptual (visual) concepts or perceived global structural configurations (see Arnheim, 1954, Chap. 2, §§ 2–5, or pp. 27–64; see also Pinna & Albertazzi, 2010), and abstraction is seen as the ability to grasp the structural elements of a type of object by its *perceived patterns*. This explains, for example, the ability to see a “house” in a configuration of toothpicks, a “face” on a wall, or a “roof” in a triangle. Structural patterns are qualitative properties of phenomena. By no means are they metrical cues.

In other words, for Gestalt psychologists, “concrete” does not denote physical objects, and “abstract” does not denote symbolic entities.

## Cross-Modality in Art Works

The field of art provides an important insight into the boundary and the relationship between concrete and abstract concepts in cross-modal phenomena (Actis Grosso et al., 2017; Albertazzi et al., 2012, 2014; Duthie, 2013; Nikolić, 2016; Noguchi, 2007; van Campen, 2010; Vergo, 2012). For example, the Western avant-garde of the 20th century was the scenario of multifarious inquiries into the perception of shape, color, and light, their expressive characteristics, and their representations in drawing and painting, in an attempt to identify and analyze their abstract dimensions (Albertazzi, Canal, Micciolo, & Vescovi, 2015; Stelzer, 1964). The switch to abstractionism and the detachment from the figurative in painting was particularly apparent in the works of Kandinsky, Mondrian, Delaunay, and Klee. The process resulted in a new conception of the theory of composition. The new tendency toward abstractionism was defined by the proponents themselves as *concrete* art (see similarities and differences of the concept in van Doesburg, 1930 and Kandinsky, 1912). The concrete/abstract couplet referred to the analysis of the subjective and emotional experience of shape and color in their rich appearances and expressivity in the visual field, devoid of any trait of objectivity (on the concept of abstract, see Kandinsky, 1912, 2013). The concrete experience expressed in painting and considered to be the essence of abstract art (Kandinsky, 1912) had no logical, syntactic, or formalized meaning: Kandinsky termed it the “spiritual” (*das Geistige*), by which he meant the subjective qualitative content (*Inhalt*) of awareness, and what he called its inner sonority/sound (*Klang*). Kandinsky’s first abstract painting (*Untitled watercolor*) dated back to 1910 and may be considered the starting point of the process (although it may appear unrelated to other paintings of the same period, see Brisch, 1955, p. 255; see also Robbins, 1963).

Kandinsky’s turn to abstractionism was prompted by a series of impressions on the nature and the interaction of color and light: For example, the connotative characteristics of colors (being rowdy, pompous, pensive, dreamy, absorbed, serious, mischievous, etc.) emitting a musical call (Kandinsky, 1912). Then, most of the cross-modal associations between vision and acoustics aroused by listening to Wagner’s *Lohengrin* in St. Petersburg and to Schönberg’s Op. 10 and Op. 11 in Munich (Göbel, 2013). These experiences were the grounds for Kandinsky’s series of *Impressions, Improvisations,* and *Compositions* paintings, starting in the 1910s.

The preparatory studies for the final works are examples of the procedure to represent the visual object in abstract form. Consider, for example, the studies for *Sketch for Composition 2*, where the process of sensible abstraction emerges in the horse-rider pattern (repeatedly drawn during the 1909 in different paintings), and *Improvisation. Gorge* (1914) representing a chaotic environment in red and yellows. Kandinsky’s first steps into abstractionism, although embedded with symbolism (such as the themes of Saint George and the dragon, the Horsemen of the Apocalypse, or the Biblical deluge), is deeply iconic (on the Gestalt process of abstraction, see earlier).

The contact and the following acquaintance and correspondence between Kandinsky and Schönberg (a painter himself) arose from the aforementioned concert in Munich and show the parallel development of inquiry into the cross-modality of color, shape, and sound in both painting and music composition (Hahl-Koch, 1984; von Maur, 1985). It was Kandinsky’s opinion that Schönberg’s music takes us into a new region where musical experiences are not acoustic but psychic (Kandinsky, 1912, Chap. 3). The affinity between Kandinsky and Schönberg is most evident during the initial period that led them to abstractionism in arts. However, while synesthesia seems to be a distinguishing trait in the painter (Ione & Tyler, 2003, pp. 223–224; van Campen, 2010, p. 56) and probably genuine and inborn if considered in the light of contemporary diagnostic criteria (Just, 2017); for a contrary opinion, see Baron-Cohen & Harrison, 1997, p. 10; Dann, 1998, p. 47; Harrison, 2001, p. 129), it is less traceable in the composer, with a few exceptions as in the case of his Op. 18, the *Die Glückliche Hand* (1910–1913). Therefore, the comparison between the two artists primarily concerns the similarity of cross-modal dimensions expressed by their works of art. Kandinsky represented the concrete experience lived by listening to Schönberg’s *Streichquartett*, Op. 10, and the *Klavierstücke*, Op. 11, in his painting *Impression 3* (*Concert*). It was Kandinsky’s opinion that Schönberg’s works, in music and painting as well, were dictated solely by the interest in the rules of organization of the *inner sound* (Kandinsky, 1912, p. 169 ff, 162).

The comparison between the two artists from a cross-modal viewpoint is possible when considering the works of the early Schönberg, which have been traditionally associated with the expressionist movement in music. This association is further supported by his own activity as a painter. In fact, although Schönberg never declared any intention to represent his music by visual art, his painting, that aroused Kandinsky’s interest, displays many expressionistic characteristics (Adams, 1995).

Whether the two different paths pursued by the two artists toward their personal form of abstractionism on similar cross-modal dimensions would be ultimately *perceivable* (i.e., not only understandable in purely intellectual terms) and associable by naïve participants was part of our research question. The occurrence of systematic associations between abstract stimuli presented in both visual and acoustic modalities, as well as their patterns, if confirmed, would shed light on the relation between the different concepts of abstraction used by the two artists; most of all, this result would contribute to the current debate on the presence of cross-modal Gestalten in highly complex stimuli.

## The Study

The study tests in one experiment divided into two phases the existence of naturally biased associations among mostly naïve participants between a series of Kandinsky’s paintings and a series of Schönberg’s musical selections. The reason for choosing these stimuli is partially the strong cross-modal experience motivating Kandinsky’s painting and its declared relationship with Schönberg’s musical innovations. The research focused on the different levels of abstraction and cross-modal associations embedded in the stimuli. The driving question was whether Kandinsky’s paintings and Schönberg’s musical pieces (those tested in the experiment) shared parallel paths toward abstractionism, as has sometimes been speculated on the basis of the facts explained in the first section: the painter through *Impressions* to *Improvisations* to *Compositions*; the composer through atonality to dodecaphony. The parallel path toward abstractionism in the two artists cannot be mapped on exactly the same time span (as mentioned, Kandinsky’s change of perspective is visible since the 1910s, Schönberg’s from the 1920s) but on a few common structural patterns expressing the new viewpoint in the two artistic fields. Also, as mentioned, Kandinsky’s reported synesthetic experience is only slightly comparable to Schönberg’s (1950). Nevertheless, for the reasons explained in the first section, it is reasonable to assume that works from the expressionist periods of both artists would be associated. As to the later periods, it is interesting to test whether the parallel drawn between the abstraction process of the two artists is accessible to participants without a theoretical knowledge of the issue. Furthermore, to study whether or not specific fragments from Schönberg’s Op. 25 can be systematically associated with Kandinsky’s paintings may shed light on the issue of the accessibility of 12-tone music by listeners as well on the idea of abstraction applied to Schönberg’s method.

As regards Kandinsky’s work, we analyzed a series of paintings dating to the years of his first *Compositions*, and his progressive detachment from the figurative representation of objects characterizing the previous Murnau phase, which resulted in painting the pure relationships among the elements of visual, cross-modal appearances. We first analyzed 25 paintings, dated from 1910 to 1923, from which we chose 15, where the progression toward abstractionism was more visible: Precisely, 14 paintings belonging to the years between 1910 and 1914 (the so-called abstract period) and 1 in 1923 (the so-called Bauhaus period; see later). The choice fell on these pictures for the following reasons. As to the shape configuration, we chose paintings showing the transition from figurality to abstractionism (representations of elementary components of pictorial space such as lines and color spots—as occurs, for example, in *Improvisation 19*, *Improvisation. Gorge*, and *Lake boat trip.* The paintings of this period are characterized by the dominance of part/whole organization according to color spots, or lines grouping, orientation, and organization; as in *Composition 5, Composition 6*, and *Improvisation 26*; horizontality and verticality (as in *Improvisation 19*, *Improvisation 14*, and *Black spot*); rhythm, such as static or dynamic (as in *Improvisation. Gorge*, *Improvisation 14*, and *Composition 7)*; connotative dimensions of color, such as bright/gloomy and fiery/faded, attractive/repulsive (as in *Composition 7, Lake boat trip*, and *Improvisation 14*); and so on. These dimensions were considered in accordance with a series of observations made by Kandinsky himself in his works (Kandinsky, 1912, Chap. V; 1913, p. 3): For instance, the structural presence of contraries (*Gegensätze*) ruling the configurations in these paintings concern mass versus line, shape versus color, warm versus cold, dark versus bright, thin versus thick, calm versus agitated, silent versus noisy, and so on. Kandinsky (1913) observes, for example, that in his *Composition 4* (1911) light-sweet-cold is the main opposition of contraries ruling the configuration of the painting (p. 35).

As regards Schönberg’s work, a total of 14 musical excerpts were extracted from his Op. 11 (*Three Piano Pieces;* Schönberg, 1990a) and Op. 25 (*Suite for Piano;* Schönberg, 1990b). Eight musical excerpts were extracted from Op. 11, and six musical excerpts from Op. 25. Musical excerpts were selected with the purpose of choosing the most representative musical passages from the two works of the composer (partly drawing on the existing literature, see comments on the individual musical excerpts in the Supplemental Material; Supko, 2017; Mayhew, 1962; Haimo, 1990), and in particular where the stylistic differences between the two different periods are most evident. For example, we selected the musical excerpts from Op. 11 trying to choose some of those passages where reminiscences of tonal harmony can be heard, and where the articulation and the agogics (intentional deviations from the basic tempo) can be distinctively attributed to the expressionist period. Similarly, we selected passages from Op. 25 where the dance features of the Baroque Suite are mostly evident in rhythm and tempo, and the ones that included the most remarkable presentations of the 12-tone material. Importantly, we tried to select musical excerpts so that each would be a complete fragment (i.e., with a perceivable beginning and end) and by trying to diversify the character of the musical excerpts as much as possible. To keep the stimuli as homogeneous as possible, the musical excerpts were extracted from recordings of the same performer (Maurizio Pollini) and were also selected to have similar durations.

Among the ones from Op. 11, three musical excerpts were chosen from the first piece (*Mäßig*), three from the second piece (*Sehr langsam*), and two from the last one (*Bewegt*). Among those from Op. 25, one musical excerpt was chosen from each movement of the *Suite*, except for the *Praeludium* (because of the impossibility to isolate a fragment from this movement that would correspond to our requirements).

As we were not testing a one-to-one correspondence between paintings and musical excerpts, it was not necessary to have an identical number of visual (15) and auditory stimuli (14).

Among Kandinsky’s production, there were many paintings that could fit our selection criteria, and we tried to include as many of them as possible (15) within reasonable task efficiency/sample size limits. Instead, the limited extension of the chosen Schönberg works, combined with our selection criteria, allowed the extraction of no more than 14 stimuli (1 less than the paintings).

The putative characteristics attributed to paintings and musical excerpts were tested in the first phase of the experiment by means of the Osgood (1956) semantic differential. The use of the Osgood semantic differential to test the associations was also partially suggested by Kandinsky’s experience of the pervasive presence of contraries in visual appearances. Kandinsky (1913), in fact, speaks of ‘fights among tones’ [*sic*], ‘contraries of the contradictions (*Widersprüche*),’ such as light/dark and their cross-modal associations (light and sweet; cold and bitter) as founding the color harmony. The specific choice of the pairs of contraries for the Osgood semantic differential, instead, besides Kandinsky’s observations, was due to features characterizing both the visual and acoustic stimuli. For example, we assumed that the calm/agitated couple might be present in a few musical excerpts, such as no. 3, no. 5, no. 6, no. 7 (agitated), and the attribute calm in the other musical excerpts taken from his Op. 11, such as musical excerpt no. 1 or no. 4 (see later). Similarly, these characteristics might be perceivable in Kandinsky’s paintings *Lake boat trip* (calm) and *Improvisation. Gorge* (agitated). The grave/acuto (left in Italian) couple was chosen to associate register and color lightness; the pair hot/cold was chosen for its potential to map color to timbres and harmonies; the pair consonant/dissonant could map subject’s perception of dissonance and roughness (see Tramo et al., 2001) of the harmony and contrasting colors in the paintings; and the pair pleasant/unpleasant as general affordances of the concrete experience of the stimuli (and not as an individual preference), as perceivable in *Black spot*, *Improvisation 3 (Concert)*, *Composition 8,* and so on.

The prediction was that the participants would make systematic associations between the paintings and the series of musical selections; and that the associations would be due to the presence of similar dimensions in both, as also to be evidenced by the Osgood semantic differential. Needless to say, both the stimuli showed a high level of complexity due to the contents of the paintings and of the musical pieces. We were also aware that, for this reason, the associations could have been conveyed by different components of the artistic works, and that it was very unlikely that we would find a one-to-one correspondence between the individual elements of the pictorial and acoustic compositions. Finally, we did not test individual preferences: Our goal was to highlight, as far as possible, the role played by the (*Gestalt*) concrete and abstract dimensions in perceiving.

## Methods

### Participants

A total of 32 participants volunteered for the two phases of the experiment: 14 women (mean age: 33.5 years; standard deviation: 16.2 years; median: 23 years) and 18 men (mean age: 40.7 years; standard deviation: 17.4 years; median: 40 years). The choice of sample size is explained in the ‘Statistical Methods’ section.

Almost all participants were recruited from students in the Departments of Humanities, Psychology and Cognitive Science, Information Engineering and Computer Science, and Mathematics, University of Trento, Italy, although we also invited some colleagues of those departments to participate in the experiment. In so doing, we had participants of different background, age, and expertise. The address list of the students was provided by the student office. We first sent an email asking the students to take part in the experiment, mentioning that we were not looking for persons with professional experience in painting and music, although four participants had received musical training at a conservatory or from private lessons, and two of them were musicians. The questionnaire reported this information. The participants were also asked about possible conscious synesthesia (Albertazzi et al., 2013; Albertazzi, Canal, & Micciolo, 2015; Palmer & Schloss, 2010; Palmer et al., 2013) and only one declared to be a synesthete. We did not include synesthesia among the exclusion criteria, as the aim was to evaluate the existence of naturally biased associations in the general population. According to Sagiv and Ward (2006), the prevalence of synesthesia could be one in 20. The only exclusion criterion was visual impairment and self-reported acoustic impairment. Before the experiment, the participants carried out the Ishihara test for color. After the experiment, the participants were asked whether they had previously known the paintings and the musical excerpts that they evaluated. For all the participants (with exception of two), the musical stimuli were totally new. A few of them were generally acquainted with one painting only (*Composition 8*).

All the participants signed an informed consent form. The two phases of the experiment reported here complied with the ethical guidelines of the University of Trento.

### Stimuli and Apparatus

The two phases of the experiment were carried on in the Experimental Phenomenology Laboratory (LabExP) at the Department of Humanities, Trento University. The laboratory had constant and controlled lighting conditions (ca. 10 lx on average in the room, given by a halogen lamp). The colors were produced on a monitor Eizo Color edge mod. CG276 (68 × 27 cm), P7N OFTD1846 75Q, Mfd. 2013.05.24, S/N 23816053 A (resolution 2560 × 1440).

The software used to calibrate the monitor was Eizo Corporation, Color Navigator 6 v.6.4.0.5. The calibration guaranteed a white D65, a 2.2 gamma, 120 cd/m^2^ luminosity, maximum contrast. The monitor was recalibrated at the beginning of each session. The auditory stimuli were administered through Sennheiser RS180 Precision Headphones.

The two phases of the experiment were performed with an interval of about 30 minutes in which the participants left the laboratory for a short rest.

## Phase 1 of the Experiment – evaluations by Osgood semantic differential

The first phase aimed to verify whether complex images and musical excerpts with varying perceptual characteristics led to consistent associations relative to a series of adjectives.

Participants were asked to rate 11 pairs of contraries (shown in random order) on a pseudo-continuous bipolar scale (ranging from 0 to 100) when looking at a painting or when listening to a musical excerpt (both presented in random order). The participants were told that they were going to be shown a set of paintings, one at a time, appearing on the left half of the screen, that could be freely zoomed by using a magnifier. The task was to evaluate the overall content (i.e., the meaning) of each painting (or of each musical excerpt) according to the 11 pairs of contraries (see later, Procedure). Pairs were presented on the right half of the screen and randomized with respect to their order of presentation in the couple (e.g., pleasant/unpleasant might be presented as unpleasant/pleasant).

The two contraries were placed at the extremes of a continuous line. Using a mouse, the participant had to place the pointer indicating his or her degree of agreement with the two adjectives with regard to the semantic content of the painting or of the musical excerpt. The software stored the choice with two scores, one for each member of the pair. Participants were allowed to change their choices at any time until they proceeded to the next stimulus. After each answer, confirmed by pressing the button ‘Confirm’ placed below the screen, a button with the inscription ‘Proceed’ appeared at the right top of the screen; by pressing it, the answer was registered and a new image or musical excerpt was presented.

### General Materials

The stimuli consisted of 15 paintings by Kandinsky and 14 musical excerpts from Maurizio Pollini’s performance extracted from Schönberg’s work (each excerpt lasted 22 seconds on average).

Kandinsky’s paintings were as follows: no. 1. *Improvisation 10* (1910); no. 2. *First abstract watercolor* (1910); no. 3. *Lake boat trip* (1910); no. 4. *Improvisation 14* (1910); no. 5. *Sketch for Composition 2* (1910); no. 6. *Composition 5* (1911); no. 7. *Improvisation 19* (1911); no. 8. *Impression 3* (*Concert*; 1911); no. 9. *Black spot* (1912); no. 10. *Landscape with red spots* (1913); no. 11. *Improvisation 26* (*Rowing*; 1912); no. 12. *Picture with a white border* (1913); no. 13. *Composition 6* (1913); no. 14. *Improvisation. Gorge* (1914); and no. 15. *Composition 8* (1923). The images were taken from the website: https://www.wassilykandinsky.net/work-150.

Schönberg’ s musical pieces were musical excerpts from Maurizio Pollini’s performance of Schönberg’s Op. 11 and Op. 25, and specifically:

*Three Piano Pieces*, Op. 11: no. 1. *Mäßig* (bars 1–8); no. 2. *Mäßig* (bars 34–41); no. 3. *Mäßig* (bars 53–58); no. 4. *Sehr langsam* (bars 2–5); no. 5. *Sehr langsam* (bars 10–13); no. 6. *Sehr langsam* (bars 43–47); no. 7. *Bewegt* (bars 1–5); and no. 8. *Bewegt* (bars 32–35).

*Suite for Piano*, Op. 25: no. 9. *Gavotte* (bars 8–16); no. 10. *Musette* (bars 1–9); no. 11. *Intermezzo* (bars 1–3); no. 12. *Menuett* (bars 1–7); no. 13. *Trio* (bars 34–42); and no. 14. *Gigue* (bars 1–13).

(The musical excerpts were produced by Iacopo Hachen).

The 11 pairs of adjectives presented in Italian were as follows: piacevole/spiacevole (pleasant/unpleasant), sereno/angoscioso (serene/distressing), grave/acuto (left in Italian, see also Albertazzi, Canal, et al., 2016), consonante/dissonante (consonant/dissonant), caldo/freddo (warm/cold), calmo/agitato (calm/agitated), acceso/spento (fiery/faded), morbido/duro (soft/hard), luminoso/cupo (bright/gloomy), statico/dinamico (static/dynamic), and dolce/aspro (sweet/bitter). Judgments were given on a continuous scale as described later.

### Procedure

The first phase of the experiment was performed using the semantic differential on a bipolar rating scale of adjectives (Osgood, 1956). The second phase of the experiment evaluated the direct association between visual and auditory stimuli.

### Task of Phase 1

Participants read the following instructions on the screen:

This experiment consists in the evaluation of visual stimuli (pictures) and auditory stimuli (musical excerpts).

On the left of the screen are the stimuli (see a painting or listen to a musical excerpt), while on the right there is a rating scale between two opposing adjectives. The cursor can be dragged to the right or left between the two adjectives, bringing it closer to one of the two. The task is to evaluate which of the two adjectives is associable with the stimulus presented, and to what extent, by positioning the cursor at the point that you consider most appropriate. You should prefer accuracy to promptness of response.

Once you have made your choice, press the “Confirm” button.

If you decide that neither of the two adjectives is associated with the stimulus, you can leave the cursor at the center and confirm the choice. By pressing the “Continue” button you can move to the evaluation of the stimulus on other pairs of opposing adjectives.

At the end of the session there will be a 30-minute break, after which the second session will start with instructions for the task to be performed.

When you are ready, press the “Start” button.

## Phase 2 of the Experiment – direct association between paintings and musical excerpts

The aim of the second phase of the experiment was to verify whether some images of Kandinsky’s paintings, with varying perceptual characteristics and contents, led to consistent cross-modal associations with musical excerpts taken from the repertoire of Schönberg’s works. Each participant saw a series of images of paintings in thumbnail on the screen (anytime the position of the paintings was randomly selected). The participant clicked on a specific image, which thus appeared in full screen mode, and likewise with the other images, without any constrained order. The participant viewed the images while simultaneously listening to a musical excerpt (presented in random order). The participant had to choose the image(s) that she or he most naturally associated with that music. She or he could choose up to three paintings associated with each musical excerpt and drag them in three different boxes at the bottom of the screen. The participant could go back to view the images again, and she or he could also listen repeatedly to the musical excerpt by pressing a button. Once the association had been performed, the selected images were moved down, each into one of the three boxes. Once the choice had been confirmed, it could not be changed, and the experiment continued with the presentation of all the images and the new musical excerpt, and so on until the musical excerpts were exhausted.

### General Materials

The stimuli consisted of the same 15 paintings by Kandinsky and 14 musical excerpts extracted from Schönberg’s work.

### Procedure

Participants read the following instructions on the screen:

You will see a series of images of paintings in thumbnail on the screen. Click on one of them, which will appear in full screen mode, and then do likewise with the other images. At the same time, you will hear a musical excerpt. Select up to three images you most naturally associate with the music, placing them in three different boxes at the bottom of the screen. You can go back to re-view images already seen, and also to hear the musical excerpt again. Once you have confirmed your choice, it cannot be changed, and the experiment will continue with further musical excerpts until there are none left. You should prefer accuracy to promptness of response.

### Statistical Methods

Descriptive statistics were calculated based on the rating values given by the participants. Euclidean distances between paintings and musical excerpts based on mean rating values for each adjective were calculated. The direct association between painting and musical excerpts was evaluated by using the χ^2^ test and the associated standardized residuals. Analyses were performed with R 3.3.1 software (R Core Team, 2016).

While the task of Phase 1 is mainly descriptive, the task of Phase 2 evaluates (using the χ^2^ test of significance) the presence of a direct association between paintings and musical clips. We expected that such an association existed and the approximate sample size was calculated in the following way:

The contingency table which collects the results of task 2 has 15 rows and 14 columns and therefore 210 cells. Participants could choose up to three paintings for each musical excerpt that they heard. All participants choose three paintings, and therefore, there are 42 observations for each participant. The statistic to test the association between the variables ‘painting’ and ‘clip’ is asymptotically distributed (under the null hypothesis) as a χ^2^ distribution (with 182 degrees of freedom). The approximation to the true χ^2^ distribution could be considered good when the lowest expected frequency is higher than 5. To obtain the total number of observations, it is necessary to multiply the number of cells (210) by the minimum number of observations in each cell (6), thus obtaining a total of 1,260 observations. As each participant contributes with 42 observations, the minimum number of participants is 1,260/42 = 30. To account for possible drop-outs, a total number of 32 subjects was considered.

## Results

### First Phase of the Experiment

Table 1 reports the mean rating values for each adjective and for each painting given by the 32 participants (the corresponding standard deviations are reported in the Supplemental Table 1). Means range between 13 and 83. Painting no. 5 (*Sketch for Composition 2*) was considered the brightest of the 15 paintings (and therefore the least gloomy) and very fiery (and therefore not faded). Similarly, painting no. 10 (*Landscape with red spots*), painting no. 1 (*Improvisation 10*), and painting no. 15 (*Composition 8*) were evaluated as very bright. Painting no. 14 (*Improvisation. Gorge*) was considered the least static (and therefore the most dynamic); similarly, painting no. 12 (*Picture with a white border*), painting no. 13 (*Composition 6*), and painting no. 6 (*Composition 5*). Painting no. 8 (*Impression III* [*Concert*]) and painting no. 5 (*Sketch for Composition 2*) were evaluated as the fieriest. Although weaker, painting no. 1 (*Improvisation 10*) and painting no. 11 (*Improvisation 26* [*Rowing*]) were also evaluated as fiery.

**Table 1.** Mean Rating Values for Each Adjective and for Each Painting Given by the 32 Participants.

	Painting														
	1	2	3	4	5	6	7	8	9	10	11	12	13	14	15
Fiery	20	41	56	43	17	37	39	18	39	30	28	49	40	27	26
Warm	47	48	58	52	30	61	53	36	45	36	40	59	52	50	51
Calm	52	63	55	46	51	59	43	59	55	38	49	58	67	69	55
Consonant	63	52	57	38	44	52	41	54	57	41	44	49	50	53	44
Sweet	64	57	65	48	41	54	52	54	55	43	42	51	48	62	57
Grave	61	55	37	41	56	50	46	56	43	54	47	45	39	53	74
Bright	23	36	65	48	13	50	47	33	49	22	34	54	57	40	23
Soft	60	49	60	36	31	44	46	48	44	40	32	36	42	51	65
Pleasant	43	42	53	37	34	38	33	39	46	31	35	40	34	33	23
Serene	51	53	63	48	35	52	50	45	57	37	39	53	59	50	31
Static	47	71	58	47	58	71	37	55	55	40	63	72	78	83	64

*Note.* Ratings can range between 0 (*closest to the label attribute*) and 100 (*closest to the antonym*). A rating close to 50 means that the stimulus was considered “neutral” on that dimension. See ‘General Materials’ section for the correspondence between numbers and stimuli.

Table 2 reports the mean rating values for each adjective and for each musical excerpt given by the same 32 participants (the corresponding standard deviations are reported in the Supplemental Table 2). Means range between 16 and 84; therefore, musical excerpt no. 1 (*Mäßig* (bars 1–8) was considered the calmest of the 14 musical excerpts (and therefore the least agitated), while musical excerpt no. 14 (*Gigue* [bars 1–13]) was considered the least static (and therefore the most dynamic).

**Table 2.** Mean Rating Values for Each Adjective and for Each Musical Excerpt Given by the 32 Participants.

	Musical excerpt													
	1	2	3	4	5	6	7	8	9	10	11	12	13	14
Fiery	64	40	50	65	52	20	23	53	30	23	67	45	26	21
Warm	41	44	58	42	59	49	53	58	52	61	54	44	55	50
Calm	16	51	48	21	45	70	81	52	58	69	25	40	66	82
Consonant	30	44	44	36	55	46	71	46	45	53	45	44	61	65
Sweet	31	44	50	39	60	56	76	60	47	68	40	35	59	63
Grave	40	45	36	27	41	65	42	22	58	72	43	58	61	63
Bright	58	47	70	74	63	35	58	75	43	41	61	45	41	39
Soft	26	41	56	31	60	52	74	60	42	61	31	37	65	68
Pleasant	30	40	42	34	48	31	54	46	33	49	32	32	37	42
Serene	34	52	63	53	63	54	69	70	42	55	51	35	51	64
Static	28	67	50	33	41	79	81	54	65	69	31	58	73	84

*Note.* Ratings can range between 0 (*closest to the label attribute*) and 100 (*closest to the antonym*). A rating close to 50 means that the stimulus was considered “neutral” on that dimension. See ‘General Materials’ section for the correspondence between numbers and stimuli.

Each painting (as well as each musical excerpt) is identified by 11 values, corresponding to the 11 adjectives. For example, painting 9 is identified by the following values (reported in column 9 of Table 1):

39 45 55 57 55 43 49 44 46 57 55

The Euclidean distance was used to assess how similar the ratings attributed to paintings and musical excerpts are to each other and therefore how similar are the paintings and the musical excerpts as assessed by the semantic differential technique. As in two dimensions, the Euclidean distance measures the usual distance between two points on a plane, in n dimensions, it evaluates the distance between two “profiles” made up of the ratings attributed by the subjects to the adjectives.

However, in order to calculate the “semantic” Euclidean distance between each painting and each musical excerpt, other 11 values are needed, that is, those corresponding to the antonym, calculated as the complement to 100 (when considering painting 9 these values are 61 55 45 43 45 57 51 56 54 43 45). These distances are shown in Table 3.

**Table 3.** Euclidean Distances Between Paintings and Musical Excerpts Based on Mean Rating Values for Each Adjective.

	Musical excerpt													
	1	2	3	4	5	6	7	8	9	10	11	12	13	14
Painting														
1	135	74	97	136	84	64	95	110	64	55	118	84	52	74
2	124	38	74	119	73	41	79	86	37	47	104	63	38	57
3	120	66	37	97	30	96	77	34	82	81	89	91	76	93
4	72	35	51	69	59	82	118	72	47	88	57	43	81	109
5	119	71	120	133	122	74	139	138	61	97	121	66	89	109
6	115	34	59	107	64	50	81	72	32	54	93	59	44	68
7	77	49	52	75	52	84	117	75	53	85	59	52	79	108
8	122	52	89	123	87	48	95	105	40	58	110	64	50	72
9	105	33	51	93	49	67	82	64	47	66	82	61	57	79
10	88	64	99	105	98	86	141	120	60	99	89	50	91	119
11	97	35	87	103	93	63	119	105	35	84	92	38	73	98
12	106	33	55	95	64	68	93	67	45	73	83	59	64	85
13	122	37	60	107	74	60	80	68	49	74	101	71	60	73
14	143	54	88	137	92	27	71	97	44	47	125	79	31	45
15	137	79	110	146	108	53	116	128	55	64	127	75	56	82

*Note.* See ‘General Materials’ section for the correspondence between numbers and stimuli.

The six painting/musical excerpts couples with the lowest distances, and therefore considered semantically more similar, are painting no. 14 (*Improvisation. Gorge*) and musical excerpt no. 6 (*Sehr langsam* [bars 43–47]), painting no. 3 (*Lake boat trip*) and musical excerpt no. 5 (*Sehr langsam* [bars 10–13]), painting no. 14 (*Improvisation. Gorge*) and musical excerpt no. 13 (*Trio* [bars 34–42]), painting no. 6 (*Composition 5*) and musical excerpt no. 9 (*Gavotte* [bars 8–16]), painting no. 12 (*Picture with a white border*) and musical excerpt no. 2 (*Mäßig* (bars 34–41), and painting no. 9 (*Black spot*) and musical excerpt no. 2 (*Mäßig* (bars 34–41).

Conversely, the painting/musical excerpt couples with the highest distances (and therefore considered semantically more different) are painting no. 15 (*Composition 8*) and musical excerpt no. 4 (*Sehr langsam* [bars 2–5]), painting no. 14 (*Improvisation. Gorge*) and musical excerpt no. 1 (*Mäßig* [bars 1–8]), painting no. 10 (*Landscape with red spots*) and musical excerpt no. 7 (*Bewegt* [bars 1–5]), painting no. 5 (*Sketch for Composition 2*) and musical excerpt no. 7 (*Bewegt* [bars 1–5]), and painting no. 5 (*Sketch for Composition 2*) and musical excerpt no. 8 (*Bewegt* [bars 32–35]).

### Second Phase of the Experiment

The χ^2^ test confirmed that the association between the variables ‘painting’ and ‘musical excerpt’ could not be considered random but instead systematic (χ^2^ = 325; *df* = 182; *p* < .001). Given that in this test the lowest expected frequency was less than 5, a Monte Carlo simulation was performed to better approximate the true sample distribution of the test. It confirmed the significance of the association (*p* < .001).

As the test did not indicate which musical excerpt was associated (positively or negatively) with which painting, a residual analysis was performed. A standardized form of the residual (which behaves like a normal deviate) was used to determine whether the residual was large enough to indicate a departure from a random choice. In this case, there was only about a 5% chance that any particular standardized residual would exceed 1.96 in absolute value. When we inspected 210 cells, about 10 residuals (i.e., 5% of 210) could have been so large solely because of random variation. On the other hand, as can be seen in Table 4, there were 26 residuals greater than 1.96 in absolute value.

**Table 4.** Standardized Residuals From the Direct Association Between Paintings and Musical Excerpts.

	Musical excerpt													
	1	2	3	4	5	6	7	8	9	10	11	12	13	14
Painting														
1	−1.07	0.44	0.47	−1.07	−1.05	−0.06	−0.56	0.47	0.47	−0.06	0.01	2.07*	−0.06	−0.03
2	−1.07	−1.16	−1.90	−1.85	−1.44	0.41	−0.33	−0.74	1.95	4.27*	−1.05	0.45	0.41	2.00*
3	0.22	−1.54	3.08*	3.17*	2.79*	−0.68	0.60	1.41	−1.94	−1.94	−0.60	−0.24	−1.52	−2.76*
4	1.90	−0.08	0.42	−0.96	0.50	−1.47	−0.98	0.42	0.89	−1.00	1.94	0.44	−0.53	−1.45
5	0.47	−1.35	1.28	1.35	−0.38	−0.02	−1.31	−1.33	0.41	−0.46	0.06	1.76	−0.46	0.00
6	−0.12	0.32	0.34	−1.15	0.42	0.86	1.40	−1.19	−0.68	−0.17	0.42	0.88	−0.68	−0.66
7	1.64	0.18	1.11	3.47*	1.21	−0.25	−1.59	1.11	−2.07*	−1.61	0.75	−0.22	−1.61	−2.05*
8	1.13	0.61	0.22	−0.58	−0.98	−1.05	0.25	2.33*	−1.47	−0.21	1.59	−1.03	−0.63	−0.18
9	−1.57	−0.89	−2.37*	0.72	1.91	0.65	−0.84	1.02	0.27	1.02	−0.01	−0.46	0.27	0.30
10	1.16	1.07	−1.35	0.17	−0.80	−0.86	−0.84	0.12	1.10	−0.86	0.20	2.11*	−0.86	−0.35
11	2.99*	1.23	−0.79	0.51	−1.54	0.85	−0.76	0.03	−0.79	−1.20	1.79	−0.76	0.03	−1.58
12	−0.18	0.15	0.18	−0.60	0.70	0.61	1.06	−1.08	1.45	−1.50	0.70	−1.05	0.18	−0.63
13	−2.35*	−0.54	2.50*	0.69	−0.42	−1.26	1.79	1.37	−0.89	−1.26	−1.56	0.27	0.62	1.03
14	−2.09*	0.62	−1.34	−1.69	−0.05	1.06	2.29*	−1.74	−0.54	1.06	−2.07*	−2.51*	2.65*	4.30*
15	−0.20	1.53	−1.61	−2.49*	−1.09	1.11	−0.68	−2.52*	2.02*	3.38*	−1.56	−0.68	1.57	1.15

*Note.* “Significant” residuals are flagged with an asterisk. See ‘General Materials’ section for the correspondence between numbers and stimuli.

Overall, there were 16 residuals greater than 1.96 and 10 residuals lower than −1.96. A positive residual means that the selected musical excerpt “attracted” the corresponding painting (i.e., the painting was associated with the musical excerpt more than the average); a negative residual means that the selected musical excerpt “repelled” the corresponding painting (i.e., the painting was associated with the musical excerpt less than the average).

There were six musical excerpts which showed a strong attraction (a residual greater than 3). Musical excerpt no. 14 (*Gigue* [bars 1–13]) was strongly associated with painting no. 14 (*Improvisation. Gorge*), musical excerpt no. 10 (*Musette* [bars 1–9]) was strongly associated with painting no. 2 (*First abstract watercolor*), musical excerpt no. 4 (*Sehr langsam* [wars 2–5]) was associated with painting no. 7 (*Improvisation 19*), musical excerpt no. 10 (*Musette* [bars 1–9]) was associated with painting no. 15 (*Composition 8*), musical excerpt no. 4 (*Sehr langsam* [bars 2–5]) was associated with painting no. 3 (*Lake boat trip*), and musical excerpt no. 3 (*Mäßig* [bars 53–58]) was associated with painting no. 3 (*Lake boat trip*).

On the other hand, the negative associations were weaker. Musical excerpt no. 14 (*Gigue* [bars 1–13]) was negatively associated with painting no. 3 (*Lake boat trip*) and showed the lowest residual (−2.76). Other three couples showed quite similar residuals (−2.5): Musical excerpt no. 8 (*Bewegt* [bars 32–35]) with painting no. 15 (*Composition 8*), musical excerpt no. 12 (*Menuett* [bars 1–7]) with image no. 14 (*Improvisation. Gorge*), and musical excerpt no. 4 (*Sehr langsam* [bars 2–5]) with painting no. 15 (*Composition 8*).

## Comparing the Results of the Two Phases

In Phase 1, participants evaluated both paintings and musical excerpts using the Osgood semantic differential, and the Euclidean distances between paintings and musical excerpts were calculated. On the other hand, in Phase 2, participants directly associated three paintings with each musical excerpt and residuals were used to evaluate the association between paintings and musical excerpts. To compare the results of the two phases, the correlation coefficient between distances and residuals was calculated. As expected, distances and residuals were inversely associated (*r* = −.357; *p* < .001), that is, on average, when a painting was “near” to a musical excerpt, the corresponding residual was high and vice versa. Therefore, some degree of consistency was found between the two phases of the experiment.

## General Discussion

The research verified the existence of cross-modal associations between a series of Kandinsky’s paintings and a series of Schönberg’s music selections. Participants were for the most part (26 of the 32) without advanced knowledge/competence in music or art. The first phase tested both paintings and musical excerpts with Osgood semantic differential; the second phase tested a direct association between paintings and musical excerpts.

The prediction that the participants would make systematic associations between the paintings and the series of musical selections, notwithstanding the diversity and high complexity of the stimuli, was largely validated.

Specifically, as regards Phase 1, with Osgood semantic differential, some of our assumptions were confirmed: For example, that the adjective ‘agitated’ might characterize some Schönberg’s musical excerpts such as no. 6, no. 7, no. 10, no. 13, and no. 14 and the adjective ‘calm’ in some of the other musical excerpts taken from Op. 11 such as no. 1 and no. 4. As predicted, the adjective ‘agitated’ characterized the painting *Improvisation. Gorge* and *Composition 6*. However, surprisingly, the painting *Lake boat trip* was not perceived as ‘calm’ (i.e., neither ‘agitated’ nor “calm”), although relatively ‘calmer’ than *Improvisation. Gorge*. However, it is interesting that none of the paintings was rated as ‘calm,’ reflecting the dynamic character of all the paintings selected in this study.

*Landscape with red spots* (no. 10) was rated as the calmest among the paintings, although not extremely calm.

Regarding the assumptions that guided the choice of the couples of contraries, the results showed the following. The warm/cold pair, surprisingly, did not result closely associated with the paintings, probably due to the great variety of colors used by Kandinsky in the selected stimuli. As to the fiery/faded and bright/gloomy pairs, the ratings seem to be more diversified with respect to the paintings, although no painting was rated neither faded nor gloomy, for similar reasons as described earlier. Also, the consonant/dissonant pair was not polarized with respect to any of the paintings, reflecting perhaps the difficulty of the participants in attributing an intuitive cross-modal meaning to such technical musical terms. The consonant/dissonant pair was more useful to describe the musical excerpts, classified as fiery, hard, and dynamic with an understandable prevalence of the ‘dissonant’ rating. The pleasant/unpleasant pair was not polarized toward ‘unpleasant,’ with respect to any of the stimuli.

As regards Phase 2, the results of the direct association between musical excerpts and paintings showed the presence of both attractions and repulsions among the stimuli. On the other hand, the negative associations were weaker, reflecting the high suitability of auditory and visual stimuli to be combined together to produce different and specific *Gestalten* among very complex stimuli.

Analysis of the findings revealed that the results obtained with the two methods (the Osgood semantic differential and direct association) do not always overlap; something that we already found in a previous study of ours (Albertazzi, Canal, et al., 2016). Although the results are generally consistent, the evaluation based on the Osgood semantic differential (Phase 1) showed in specific occurrences a partial dissimilarity in the results of the evaluation based on direct association (Phase 2).

This partial diversity between the two phases of the experiment may reveal a structural difference between the linguistic and the perceptual mediums adopted in the evaluations, which is important to bear in mind in the debate among synesthesia, cross-modality, and ideasthesia. This result suggests that the semantic differential methods, also used in other related studies (e.g., Rančić & Marković, 2019), might be insufficient to fully account for cross-modal correspondences, in particular for multimodal *Gestalt* effects.

As to the aim of our study, we may conclude that the cross-modal patterns toward abstractionism, manifested in the works of the two artists, were often detectable by the participants and comparable in the first phases of their development (for Kandinsky, the period starting in the 1910s, for Schönberg the period corresponding to his development of atonal music). Participants were also able to detect the change in Schönberg’s compositional structure leading to dodecaphony, which was, however, less associable with the figural elements still remaining in the second period of Kandinsky’s painting toward abstractionism (mainly, *Compositions*). In fact, Schönberg’s dodecaphonic construction is a product of a more explicit mental operation put in place by the composer.

Concerning the debate on the opposite viewpoints in cross-modal studies, the results shed new light on the concept of ideasthesia. Ideasthesia is commonly conceived as the activation of concepts producing phenomenal experience, where concepts are generally understood in Fodorian terms, that is, as top-down cognitive abilities for our comprehension of the environment, and semantically driven. This conception, for example, equates associations between color and temperature (subjectively, red is warm, blue is cold) to associations of the type doctor and nurse (Nikolić, 2016). These concepts and associations, however, are categorically very different (and presumably mediated by different neural pathways). As Hering and Katz observed, it is in the (universal) nature of the color red to be warm and of the color blue to be cold (Da Pos & Albertazzi, 2003; Da Pos & Valenti, 2007; Hering, 1920/1964; Katz, 1935). The associations perceived between color and temperature, on which there is a general agreement in the field of arts (Albers, 1975; Kandinsky, 1912; Itten, 1961), are produced by *concrete* experience directly given in awareness. Color and its experienced temperature are not disjunctive and detachable components of a percept; moreover, they are not a product of top-down external associations between ‘abstract’ (in the sense of formal or syntactically processed) concepts due to past experience, according to the Humean and probabilistic hypothesis. They are internally related as a unitary qualitative content of awareness. Color and its experienced temperature form a unitary (*Gestalt*) concrete concept. Doctor and nurse, and similar pairs of concept associations, instead, are only externally, conventionally, and functionally related. Needless to say, the connotative properties of color, among which warmth/coldness, may be weakened by the context inducing phenomena of assimilation (see in Supplemental Material the comment on musical excerpt no. 8).

A point to be considered in the debate on ideasthesia, however, is firstly the profound symbolism of which Kandinsky’s work is imbued and its impact on the final configurations of his paintings. Strict supporters of ideasthesia, in fact, may argue that the strong cross-modal (sometimes considered as synesthetic) content of his paintings has been induced by his theosophical or religious ideation (Kandinsky, 1912, Chap. 3), and the same would hold for Schönberg. In this respect, Kandinsky’s paintings and Schönberg’s music play the role of a case study because they are both culturally imbued with ideas circulating in the avant-garde of the 20th century (Washton Long, 1972, 1975). However, as Kanizsa (1991) clearly stated, in perceptual analyses, heuristically one should distinguish phenomena pertaining to seeing (and other modalities) from phenomena pertaining to thinking. As to Kandinsky’s own descriptions of his cross-modal experience analyzed with contemporary diagnostic criteria, they might allow us to hypothesize that the phenomenon is genuinely synesthetic (see e.g., Kandinsky, 1912, p. 179). Factually, the dimensions of seeing and thinking are co-present in ideation but still distinguishable. For example, the figure of the ‘rider’ or ‘horseman,’ as frequently appears in Kandinsky paintings, from a cultural viewpoint represents the knight of the Apocalypse. However, the cultural meaning as such cannot be considered directly responsible in determining or inducing the process of sensible abstraction in Kandinsky’s works. In fact, the pattern (a rider, a horse) can appear in totally different cultural environments and contexts (such as in children’s drawings, see Arnheim, 1969, Chap. 14). Moreover, most of the participants were totally unaware of Kandinsky’s cultural and religious background, and therefore, it could not have influenced their choices.

Second, as regards the tendency to abstractionism prompted by the *Jugendstil* (*Art Nouveau*), through ornamental patterns and their intrinsic symbolism, although it influenced Kandinsky’s formation during his years of training in Munich (Stelzer, 1964; Weiss, 1975), it is not what grounded his path to abstractionism: As he observed, it was the contents of his subjective experience that drove the process that led to the series of sketches for the creation of the *Impressions* (see, e.g., *Gendarme*, *Impression 4* [1911]) to *Improvisations* (see e.g., *Improvisation 14* [1910]—grounded on the interplay between color, form, brightness, and figural elements [lines, cuspids, heights]—to *Composition*s, during the years 1911–1914; see e.g., the transition from *Landscape with tower* [1909] to *Church in Murnau* [1910]; see Kandinsky, 1912, Chap. 8).

Similarly, as to the possible influence of theosophy on Kandinsky’s painting and theory (e.g., on the German concept of “*Geist*,” which is undeniable; Kandinsky, 1912, Chap. 3), it cannot be considered the decisive top-down factor in Kandinsky’s production (for a different opinion, see Ringbom, 1966; on the diffuse interest in synesthesia by artists of the same period, see Besant & Leadbeater, 1901). Comparable influences on his theory of color were exerted by the theories of Goethe, Runge, Bezold, Wagner, Debussy, Fiedler, Swedenborg, Th. Lipps, and Worringer (e.g., the idea of a total work of art, the concept of visibility, and of inner necessity, see Kandinsky, 1912, Chap. 6). These theories, however, were diffused, discussed, and influential through the main avant-gardes of the time. The same holds for Schönberg.

Finally, as the results of our study show, the meanings responsible for the associations were neither directly language nor culture-driven: And this is certainly true in the case of the second phase of the experiment, where no language dimensions were involved.

Common patterns were perceived by the participants between Kandinsky’s first step toward abstractionism and Schönberg’s shift toward atonality. Subsequently, the composer’s artistic turn to dodecaphony, mainly driven by an intellectual process (and therefore less influenced by perceptual dimensions during the creation process), showed perceivable gaps between the two artists’ works of art. And in fact, in the case of Kandinsky, a greater concrete sensibility toward cross-modal dimensions in perceiving can be traced in his whole production. Vice versa, in Schönberg, a sensitivity of this type, as mentioned, can be traced only in a defined period.

The fact that association patterns were weaker between Schönberg’s Op. 25 and the later Kandinsky’s works could be ascribed to the (very) different notions of abstractionism that can be applied to the two artists’ creative development. In their transition between different periods, while Kandinsky’s explicit aim was to abstract the qualitative content of perception, Schönberg aimed at giving a systematic order to qualities that had already been abstracted in his (and others’) earlier works. Consider Op. 11, which in turn would contribute to inspiring Kandinsky’s detachment from the figurative. If the association between Kandinsky’s second period and Schönberg’s Op. 25 does not result from a common theoretical view, it can nevertheless result from the structural change in both artists’ works, also influenced by the historical context. The detachment of both artists from their earlier technical habits, inducing Kandinsky to develop a more systematic framework to objectify conscious experience, and Schönberg to devise a technique to systematically organize atonality, in fact, results in an increase in the methodological complexity of their works (e.g., in Kandinsky’s latest *Compositions*, such as *Composition 8*, or the series of *Improvisation*s, such as *Improvisation. Gorge*), which is incidentally perceivable to the viewer/listener (although not necessarily conceptually understandable) at least when matching the stimuli together.

Further studies might test expert musicians and painters only to verify whether the same correspondences take place and eventually to a greater extent in the case of expertise; or people from different cultures, totally unaware of Kandinsky’s and Schönberg’s works. Another possibility would be to test Kandinsky’s paintings of the Murnau period with the work of other composers such as Hartmann or Wagner. Then, assuming the presence of synesthetic traits in Kandinsky’s works of art, it would be interesting to verify the comparison between Kandinsky and Scriabin on light and color (Kandinsky, 1912). A specific study might also focus on Schönberg’s Op. 18 and some of his own paintings of the same period.

As to the choice of the pairs of contraries for the Osgood semantic differential, a further analysis might be conducted on color/shape associations limited to the aforementioned connotative properties of color specifically experienced by Kandinsky.

## Supplemental Material

sj-jpg-1-ipe-10.1177_2041669520950750 - Supplemental material for Cross-Modal Perceptual Organization in Works of Art Click here for additional data file. Supplemental material, sj-jpg-1-ipe-10.1177_2041669520950750 for Cross-Modal Perceptual Organization in Works of Art by Liliana Albertazzi, Luisa Canal, Rocco Micciolo and Iacopo Hachen in i-Perception

sj-jpg-2-ipe-10.1177_2041669520950750 - Supplemental material for Cross-Modal Perceptual Organization in Works of Art Click here for additional data file. Supplemental material, sj-jpg-2-ipe-10.1177_2041669520950750 for Cross-Modal Perceptual Organization in Works of Art by Liliana Albertazzi, Luisa Canal, Rocco Micciolo and Iacopo Hachen in i-Perception

sj-jpg-3-ipe-10.1177_2041669520950750 - Supplemental material for Cross-Modal Perceptual Organization in Works of Art Click here for additional data file. Supplemental material, sj-jpg-3-ipe-10.1177_2041669520950750 for Cross-Modal Perceptual Organization in Works of Art by Liliana Albertazzi, Luisa Canal, Rocco Micciolo and Iacopo Hachen in i-Perception

sj-jpg-4-ipe-10.1177_2041669520950750 - Supplemental material for Cross-Modal Perceptual Organization in Works of Art Click here for additional data file. Supplemental material, sj-jpg-4-ipe-10.1177_2041669520950750 for Cross-Modal Perceptual Organization in Works of Art by Liliana Albertazzi, Luisa Canal, Rocco Micciolo and Iacopo Hachen in i-Perception

sj-jpg-5-ipe-10.1177_2041669520950750 - Supplemental material for Cross-Modal Perceptual Organization in Works of Art Click here for additional data file. Supplemental material, sj-jpg-5-ipe-10.1177_2041669520950750 for Cross-Modal Perceptual Organization in Works of Art by Liliana Albertazzi, Luisa Canal, Rocco Micciolo and Iacopo Hachen in i-Perception

sj-jpg-6-ipe-10.1177_2041669520950750 - Supplemental material for Cross-Modal Perceptual Organization in Works of Art Click here for additional data file. Supplemental material, sj-jpg-6-ipe-10.1177_2041669520950750 for Cross-Modal Perceptual Organization in Works of Art by Liliana Albertazzi, Luisa Canal, Rocco Micciolo and Iacopo Hachen in i-Perception

sj-jpg-7-ipe-10.1177_2041669520950750 - Supplemental material for Cross-Modal Perceptual Organization in Works of Art Click here for additional data file. Supplemental material, sj-jpg-7-ipe-10.1177_2041669520950750 for Cross-Modal Perceptual Organization in Works of Art by Liliana Albertazzi, Luisa Canal, Rocco Micciolo and Iacopo Hachen in i-Perception

sj-jpg-8-ipe-10.1177_2041669520950750 - Supplemental material for Cross-Modal Perceptual Organization in Works of Art Click here for additional data file. Supplemental material, sj-jpg-8-ipe-10.1177_2041669520950750 for Cross-Modal Perceptual Organization in Works of Art by Liliana Albertazzi, Luisa Canal, Rocco Micciolo and Iacopo Hachen in i-Perception

sj-jpg-9-ipe-10.1177_2041669520950750 - Supplemental material for Cross-Modal Perceptual Organization in Works of Art Click here for additional data file. Supplemental material, sj-jpg-9-ipe-10.1177_2041669520950750 for Cross-Modal Perceptual Organization in Works of Art by Liliana Albertazzi, Luisa Canal, Rocco Micciolo and Iacopo Hachen in i-Perception

sj-jpg-10-ipe-10.1177_2041669520950750 - Supplemental material for Cross-Modal Perceptual Organization in Works of Art Click here for additional data file. Supplemental material, sj-jpg-10-ipe-10.1177_2041669520950750 for Cross-Modal Perceptual Organization in Works of Art by Liliana Albertazzi, Luisa Canal, Rocco Micciolo and Iacopo Hachen in i-Perception

sj-jpg-11-ipe-10.1177_2041669520950750 - Supplemental material for Cross-Modal Perceptual Organization in Works of Art Click here for additional data file. Supplemental material, sj-jpg-11-ipe-10.1177_2041669520950750 for Cross-Modal Perceptual Organization in Works of Art by Liliana Albertazzi, Luisa Canal, Rocco Micciolo and Iacopo Hachen in i-Perception

sj-jpg-12-ipe-10.1177_2041669520950750 - Supplemental material for Cross-Modal Perceptual Organization in Works of Art Click here for additional data file. Supplemental material, sj-jpg-12-ipe-10.1177_2041669520950750 for Cross-Modal Perceptual Organization in Works of Art by Liliana Albertazzi, Luisa Canal, Rocco Micciolo and Iacopo Hachen in i-Perception

sj-jpg-13-ipe-10.1177_2041669520950750 - Supplemental material for Cross-Modal Perceptual Organization in Works of Art Click here for additional data file. Supplemental material, sj-jpg-13-ipe-10.1177_2041669520950750 for Cross-Modal Perceptual Organization in Works of Art by Liliana Albertazzi, Luisa Canal, Rocco Micciolo and Iacopo Hachen in i-Perception

sj-jpg-14-ipe-10.1177_2041669520950750 - Supplemental material for Cross-Modal Perceptual Organization in Works of Art Click here for additional data file. Supplemental material, sj-jpg-14-ipe-10.1177_2041669520950750 for Cross-Modal Perceptual Organization in Works of Art by Liliana Albertazzi, Luisa Canal, Rocco Micciolo and Iacopo Hachen in i-Perception

sj-jpg-15-ipe-10.1177_2041669520950750 - Supplemental material for Cross-Modal Perceptual Organization in Works of Art Click here for additional data file. Supplemental material, sj-jpg-15-ipe-10.1177_2041669520950750 for Cross-Modal Perceptual Organization in Works of Art by Liliana Albertazzi, Luisa Canal, Rocco Micciolo and Iacopo Hachen in i-Perception

sj-pdf-16-ipe-10.1177_2041669520950750 - Supplemental material for Cross-Modal Perceptual Organization in Works of Art Click here for additional data file. Supplemental material, sj-pdf-16-ipe-10.1177_2041669520950750 for Cross-Modal Perceptual Organization in Works of Art by Liliana Albertazzi, Luisa Canal, Rocco Micciolo and Iacopo Hachen in i-Perception

sj-xlsx-17-ipe-10.1177_2041669520950750 - Supplemental material for Cross-Modal Perceptual Organization in Works of Art Click here for additional data file. Supplemental material, sj-xlsx-17-ipe-10.1177_2041669520950750 for Cross-Modal Perceptual Organization in Works of Art by Liliana Albertazzi, Luisa Canal, Rocco Micciolo and Iacopo Hachen in i-Perception
