# Supplementary figures and images for: Cross-Modal Perceptual Organization in Works of Art
Source: Iperception. 2020 Aug 27;11(4):2041669520950750. doi: 10.1177/2041669520950750 (PMC7459189; doi:10.1177/2041669520950750)

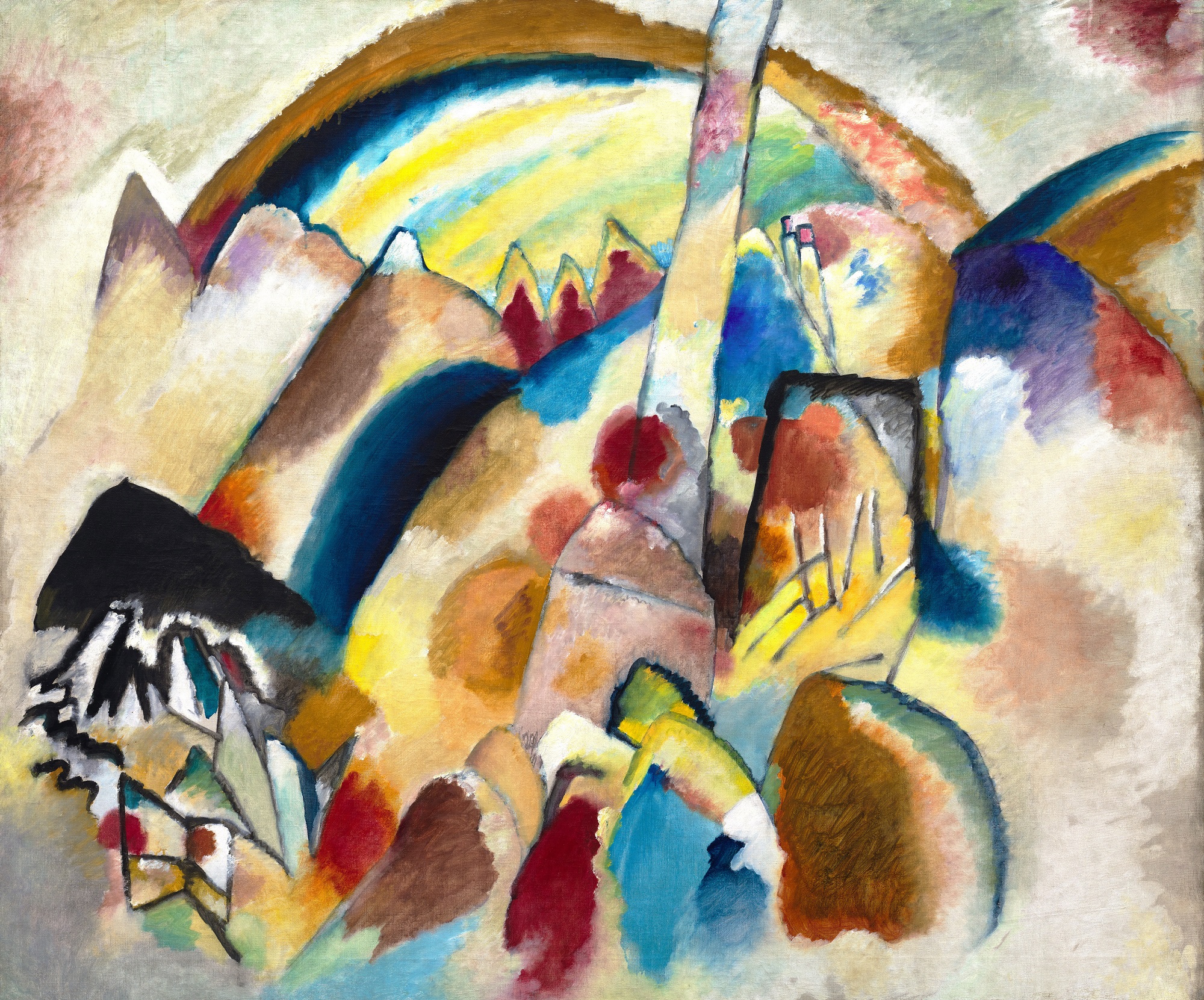

Supplement: sj-jpg-1-ipe-10.1177_2041669520950750 - Supplemental material for Cross-Modal Perceptual Organization in Works of Art [file sj-jpg-1-ipe-10.1177_2041669520950750.jpg]

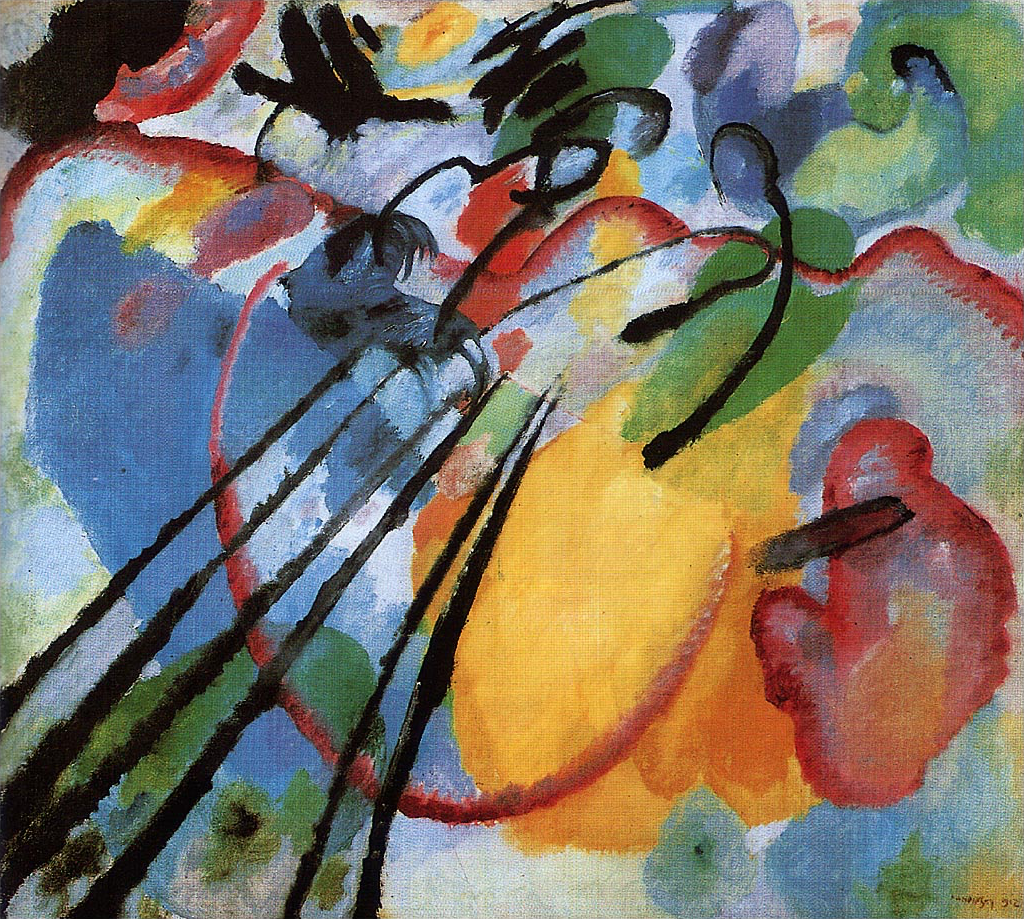

Supplement: sj-jpg-2-ipe-10.1177_2041669520950750 - Supplemental material for Cross-Modal Perceptual Organization in Works of Art [file sj-jpg-2-ipe-10.1177_2041669520950750.jpg]

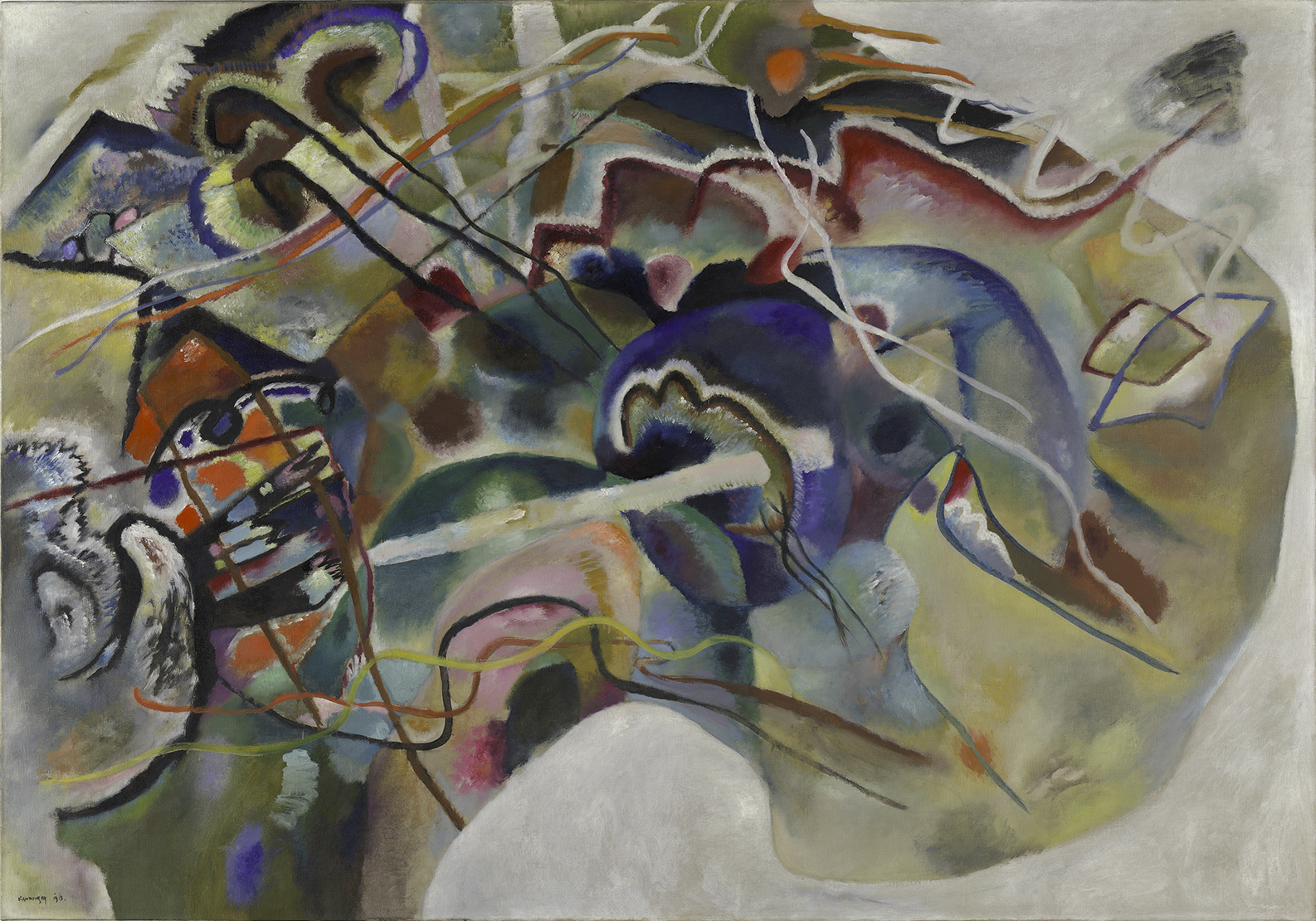

Supplement: sj-jpg-3-ipe-10.1177_2041669520950750 - Supplemental material for Cross-Modal Perceptual Organization in Works of Art [file sj-jpg-3-ipe-10.1177_2041669520950750.jpg]

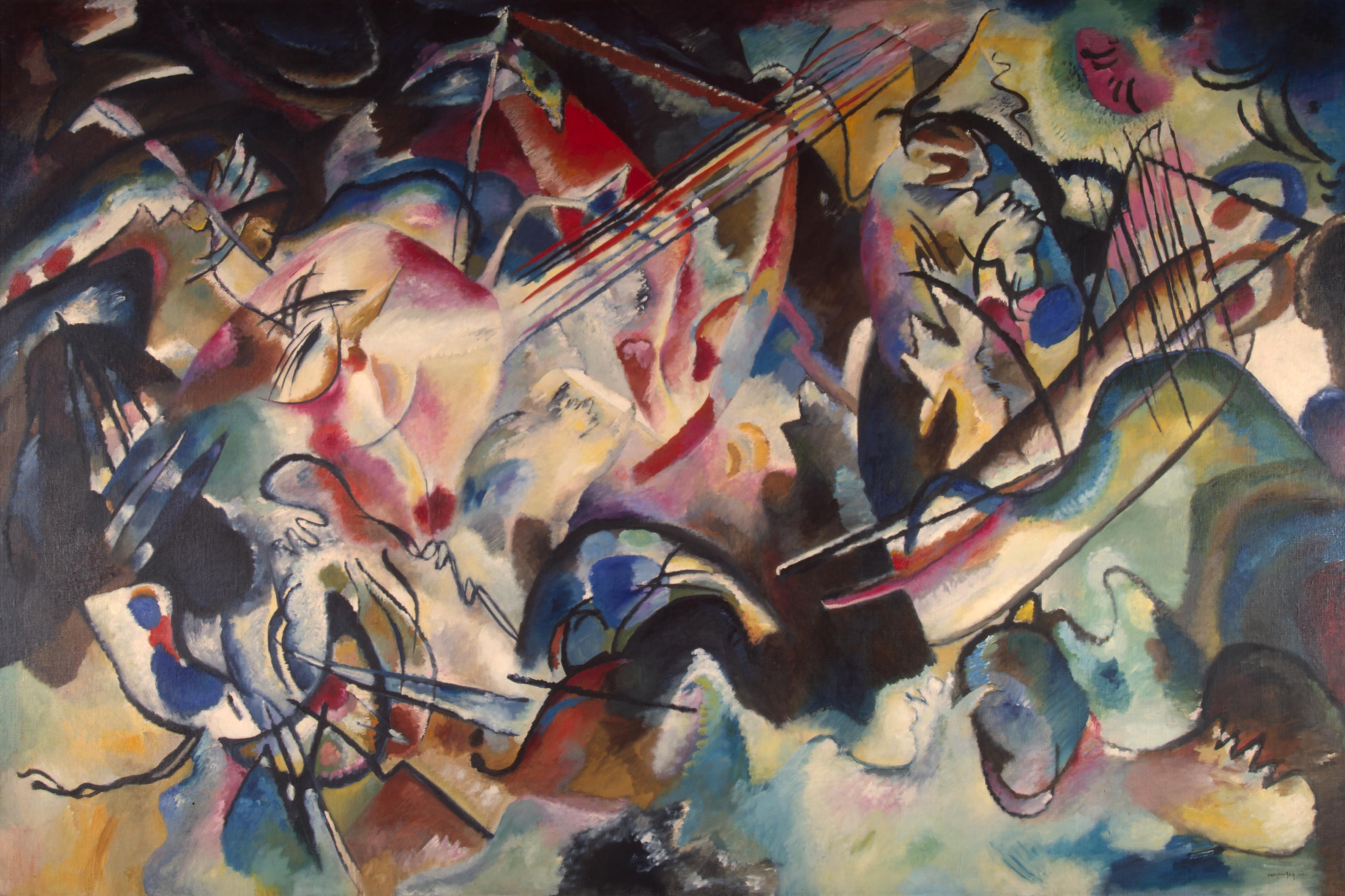

Supplement: sj-jpg-4-ipe-10.1177_2041669520950750 - Supplemental material for Cross-Modal Perceptual Organization in Works of Art [file sj-jpg-4-ipe-10.1177_2041669520950750.jpg]

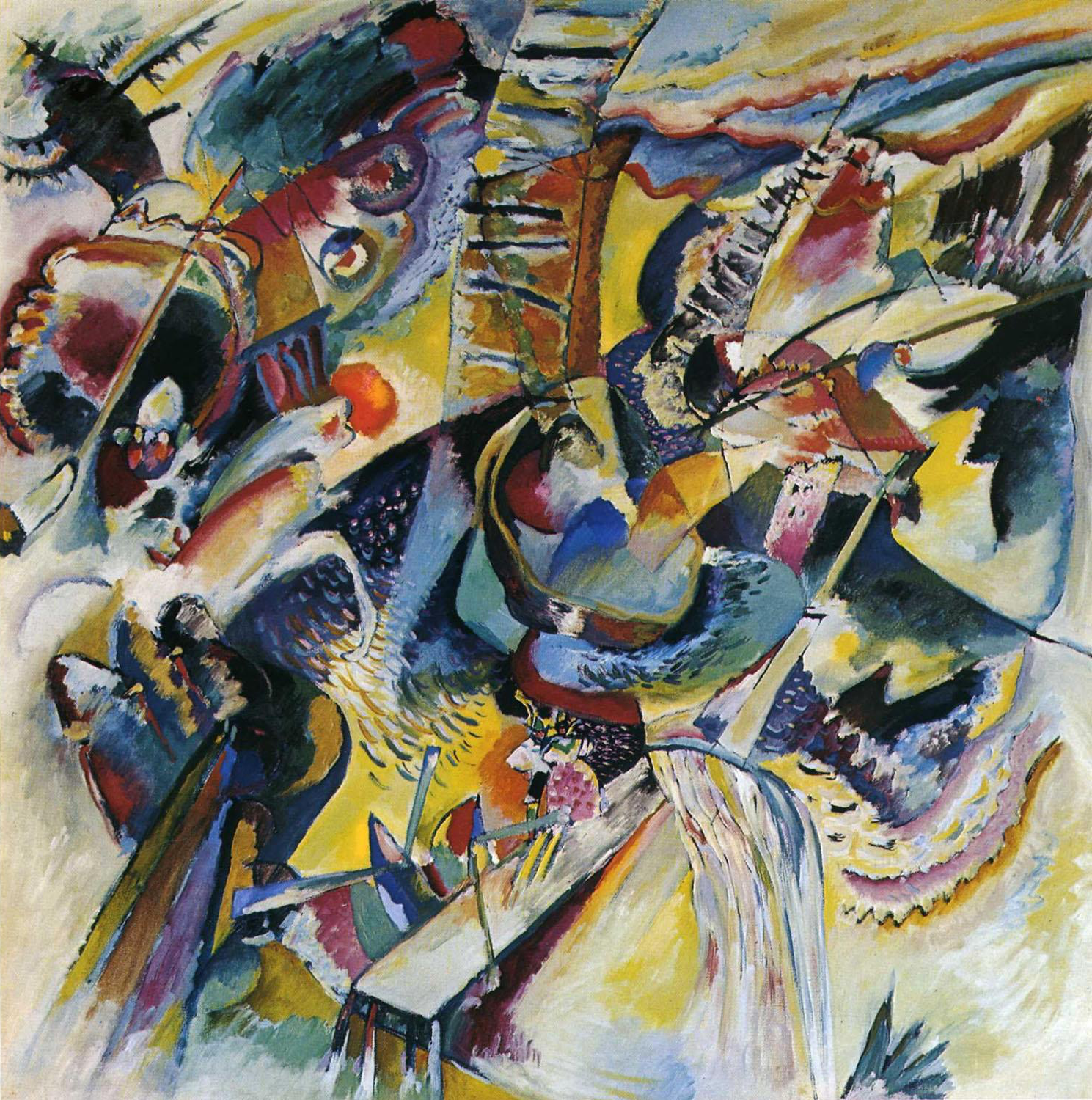

Supplement: sj-jpg-5-ipe-10.1177_2041669520950750 - Supplemental material for Cross-Modal Perceptual Organization in Works of Art [file sj-jpg-5-ipe-10.1177_2041669520950750.jpg]

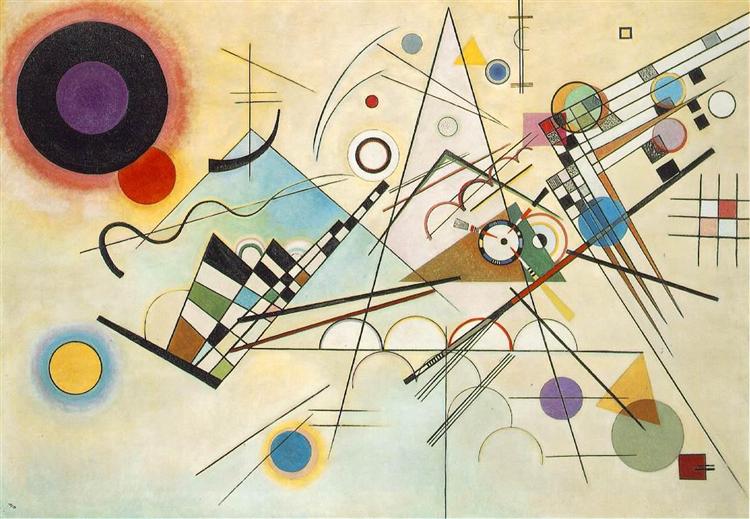

Supplement: sj-jpg-6-ipe-10.1177_2041669520950750 - Supplemental material for Cross-Modal Perceptual Organization in Works of Art [file sj-jpg-6-ipe-10.1177_2041669520950750.jpg]

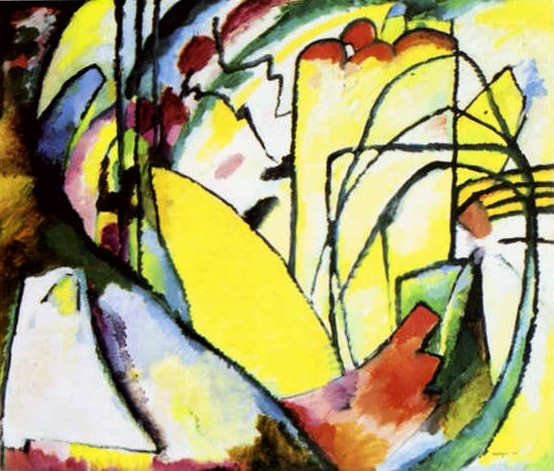

Supplement: sj-jpg-7-ipe-10.1177_2041669520950750 - Supplemental material for Cross-Modal Perceptual Organization in Works of Art [file sj-jpg-7-ipe-10.1177_2041669520950750.jpg]

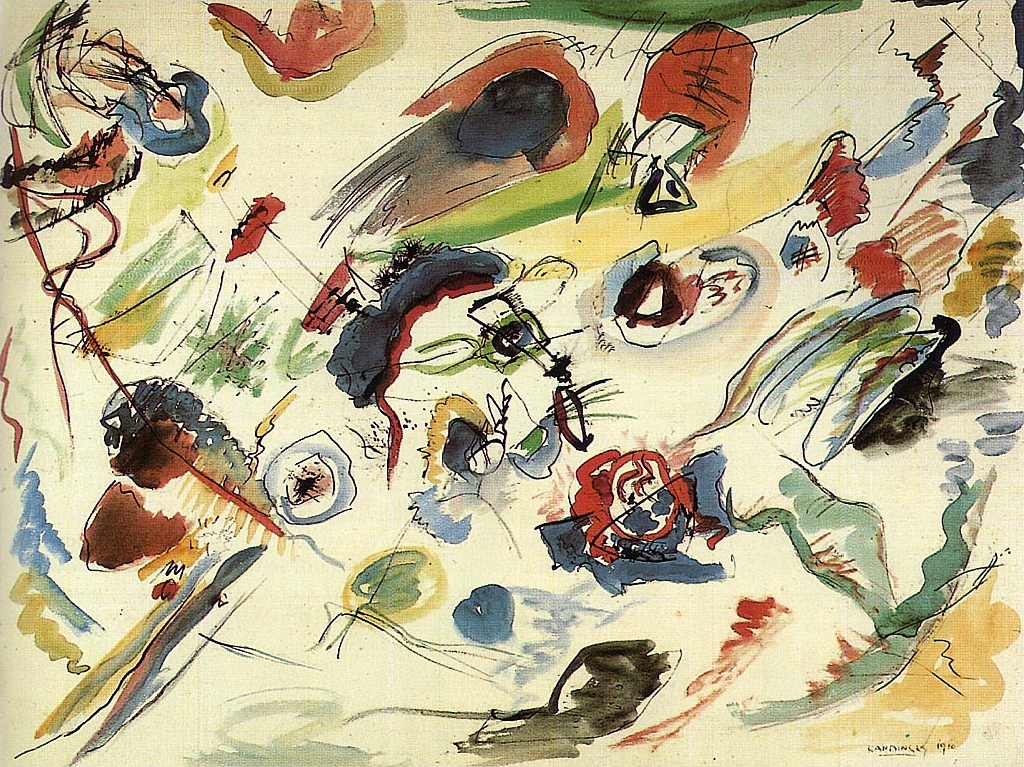

Supplement: sj-jpg-8-ipe-10.1177_2041669520950750 - Supplemental material for Cross-Modal Perceptual Organization in Works of Art [file sj-jpg-8-ipe-10.1177_2041669520950750.jpg]

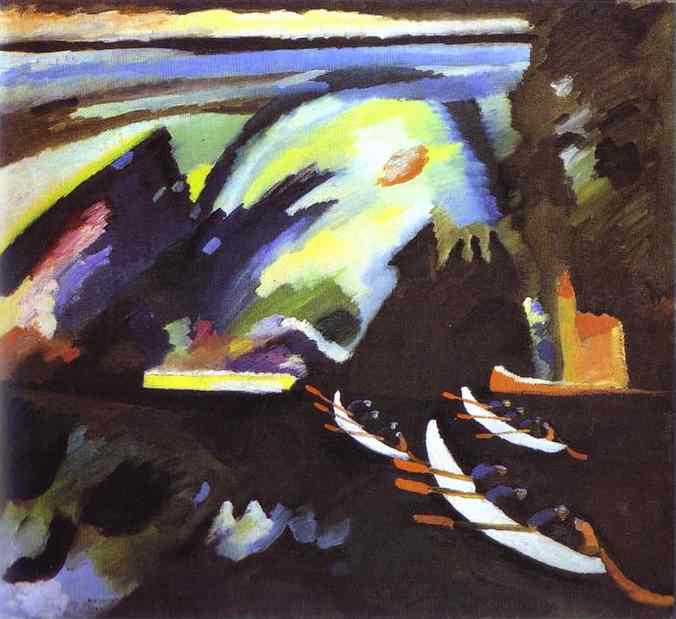

Supplement: sj-jpg-9-ipe-10.1177_2041669520950750 - Supplemental material for Cross-Modal Perceptual Organization in Works of Art [file sj-jpg-9-ipe-10.1177_2041669520950750.jpg]

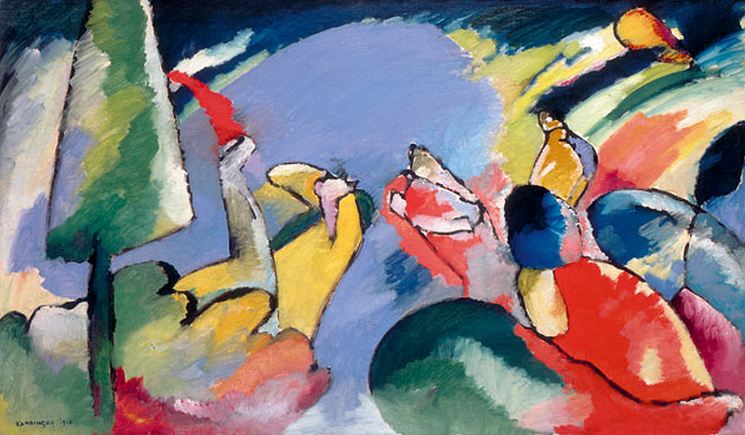

Supplement: sj-jpg-10-ipe-10.1177_2041669520950750 - Supplemental material for Cross-Modal Perceptual Organization in Works of Art [file sj-jpg-10-ipe-10.1177_2041669520950750.jpg]

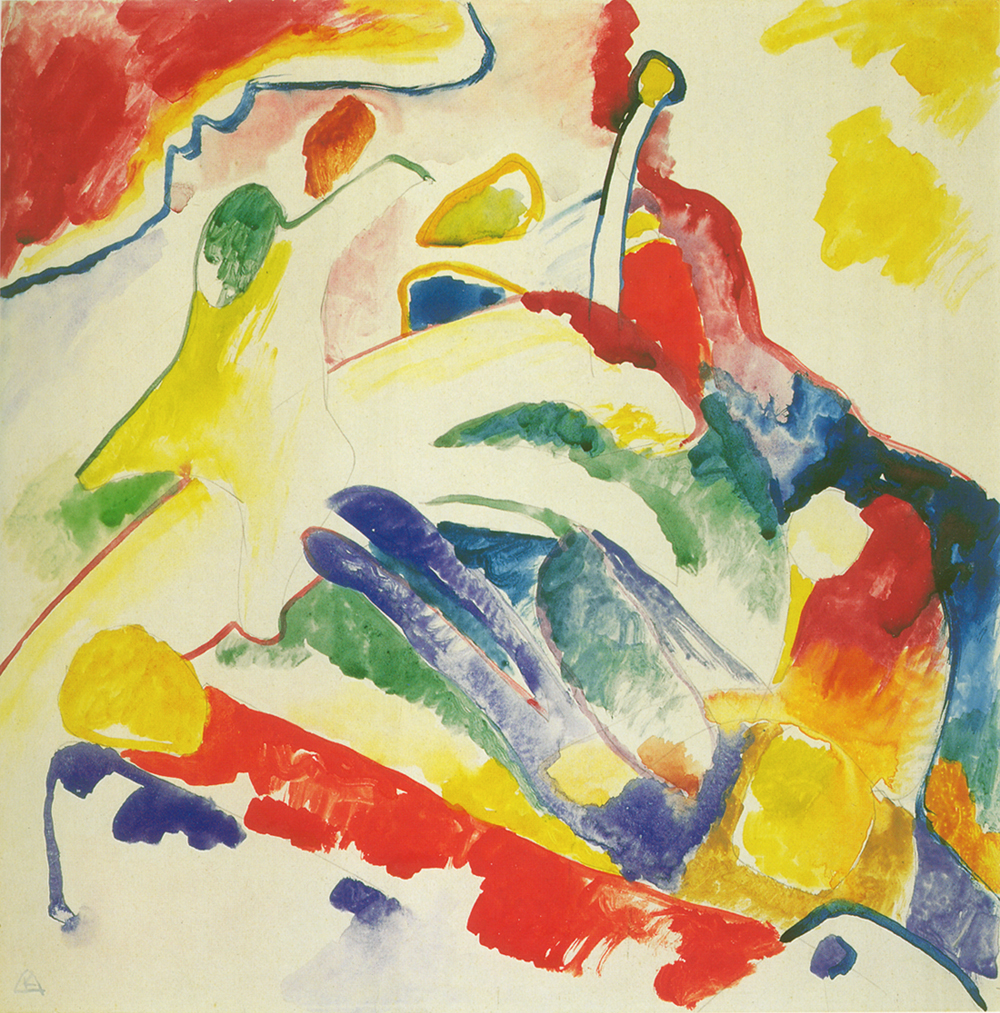

Supplement: sj-jpg-11-ipe-10.1177_2041669520950750 - Supplemental material for Cross-Modal Perceptual Organization in Works of Art [file sj-jpg-11-ipe-10.1177_2041669520950750.jpg]

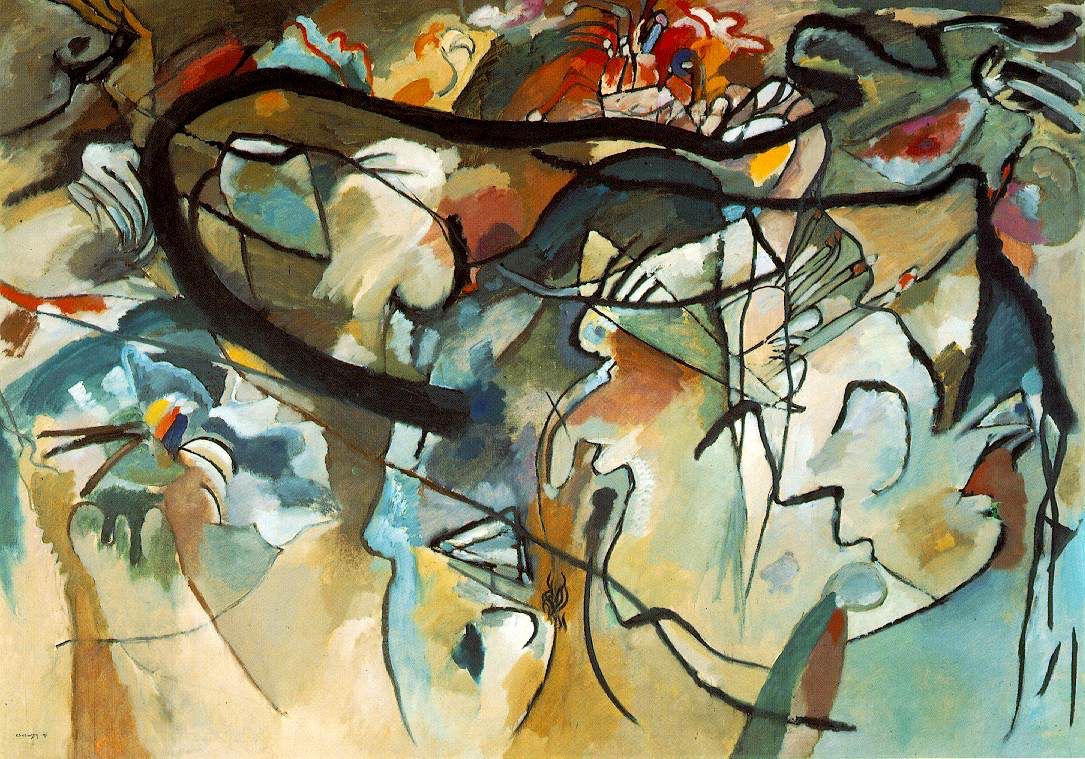

Supplement: sj-jpg-12-ipe-10.1177_2041669520950750 - Supplemental material for Cross-Modal Perceptual Organization in Works of Art [file sj-jpg-12-ipe-10.1177_2041669520950750.jpg]

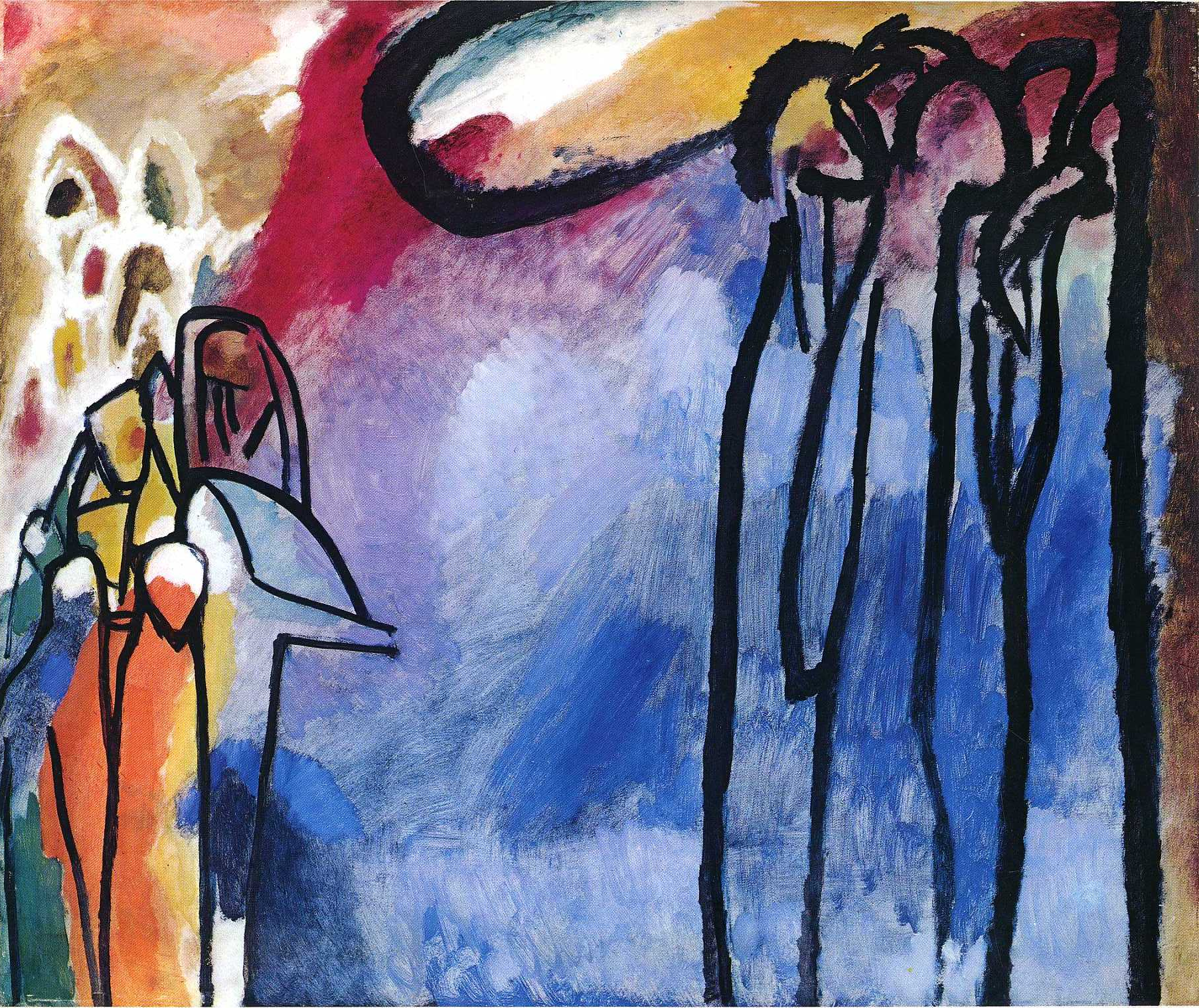

Supplement: sj-jpg-13-ipe-10.1177_2041669520950750 - Supplemental material for Cross-Modal Perceptual Organization in Works of Art [file sj-jpg-13-ipe-10.1177_2041669520950750.jpg]

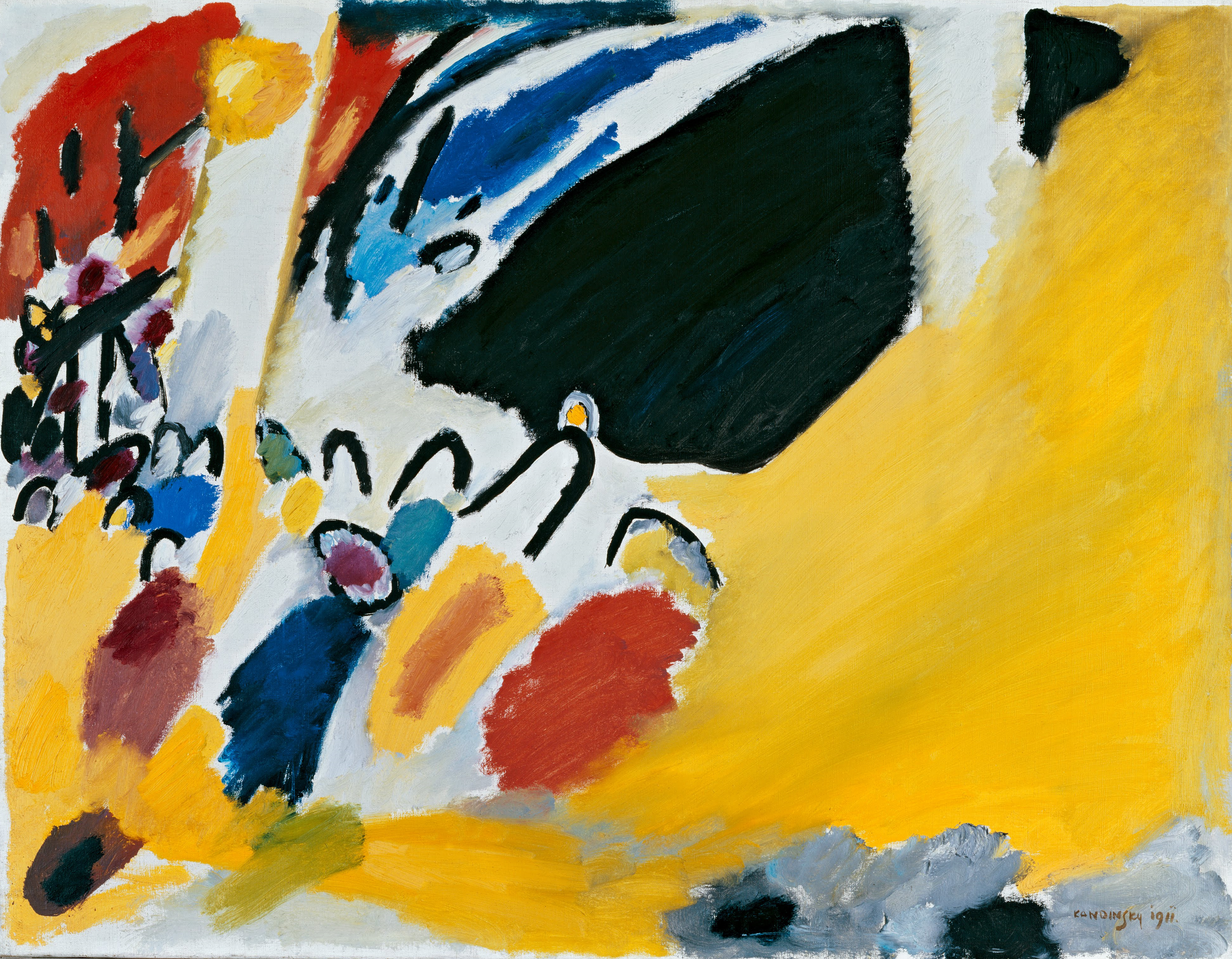

Supplement: sj-jpg-14-ipe-10.1177_2041669520950750 - Supplemental material for Cross-Modal Perceptual Organization in Works of Art [file sj-jpg-14-ipe-10.1177_2041669520950750.jpg]

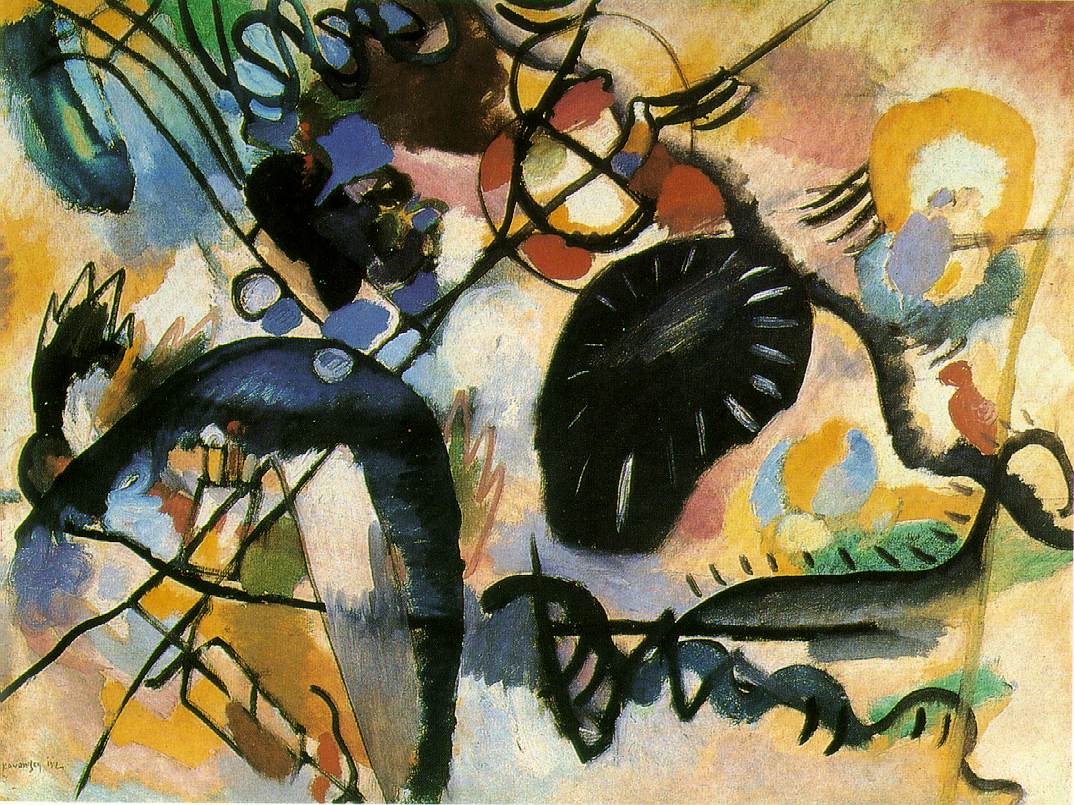

Supplement: sj-jpg-15-ipe-10.1177_2041669520950750 - Supplemental material for Cross-Modal Perceptual Organization in Works of Art [file sj-jpg-15-ipe-10.1177_2041669520950750.jpg]
