# Supplementary material for: Cross-Modal Perceptual Organization in Works of Art
Source: Iperception. 2020 Aug 27;11(4):2041669520950750. doi: 10.1177/2041669520950750 (PMC7459189; doi:10.1177/2041669520950750)
Supplement: sj-pdf-16-ipe-10.1177_2041669520950750 - Supplemental material for Cross-Modal Perceptual Organization in Works of Art [file sj-pdf-16-ipe-10.1177_2041669520950750.pdf]

Supplemental Material (Comments on the stimuli associations)

***Three Piano Pieces*, Op. 11:**

1. *Mäßig* (bars 1-8)

Osgood Differential results: With the Osgood semantic differential, Clip 01 was evaluated as calmo (calm), consonante (consonant), dolce (sweet), morbido (soft), piacevole (pleasant), sereno (serene), statico (static), in a statistically significant way.

Musical analysis: The above ratings are well suited to the character of this clip. The fragment consists in the presentation of the main theme of the first piece (*mäßig*). It shows evident tonal elements, such as for example the resolution of an augmented triad in measure number 3 (for more details, see Supko, 2017). Phrasing is smooth and somehow lyrical, involving *legato* and predominantly light dynamics (*p*). Tempo is moderate (*mäßig*), becoming even slower in the final *rallentando* at measure 9, which precedes the indication of *langsamer*. The repetition of the ascending pattern in the left hand contributes to giving the fragment a certain degree of coherence, which could have further contributed to the rating of static.

Notable associations/repulsions: In terms of Euclidean distance, this clip proves to be repelled by several of the paintings, among which *Improvisation 10*, *First abstract watercolor*, *Composition 6*, *Improvisation (Gorge)*, and *Composition 8*. These paintings seem to be all characterized by strong dynamism and the use of very bright and/or diverse colors, which seems to be incompatible with the simplicity of the fragment and its overall calm atmosphere (see ratings above).

Pictorial analysis of the associated paintings: In particular, *Composition 6* intentionally shows different and contrasting areas (smooth and rough) and contrasting colors, expressing the mood

of chaos and violence (the Deluge). For example, as Kandinsky commented (https://www.wassilykandinsky.net/work-35.php), to the right appears a rough, red-blue – a sort of dissonant center drawn with sharp, strong, precise lines; while to the left a gentle, pink - a sort of slightly blurred center drawn with faint, uncertain lines. Kandinsky also mentions his choice of long solemn lines that connect to heavy transverse lines running to them deliberately at the top of the painting and entering into a direct conflict with them.

Comparison with Direct Association results: By means of Task 2, among the repulsions of Clip 1, two of the previous paintings were also statistically significant: *Improvisation 6,* and *Improvisation (Gorge)*. Notably, the repulsion with *Composition 6* does not hold in the results of Task 2, which may be partially due to the matching of the light background with the character of the clip.

02. *Mäßig* (bars 34-41)

Osgood Differential results: With the Osgood semantic differential, no rating apart from ‘dynamic’ exceeded statistical threshold for Clip 02, an occurrence that can be explained by considering its very heterogeneous contents, making it difficult to classify univocally on the given dimensions.

Musical analysis: In the fragment, the right hand starts to play a variation of the main theme in double notes rapidly alternating with a transposed version of the same theme, resulting in a sort of arpeggio. The dynamic starts very soft (*pp-ppp*) and with fluent articulation (*fliessende*r), smoothly increasing and rising in register, together with the left-hand playing fragments of an ascending chromatic scale in broken octaves. At the apex of the *crescendo*, the main rhythmic pattern is disrupted and followed by the repetition of very different patterns with multiple ‘ups’ and ‘downs’, probably responsible for the rating of dinamico (dynamic), giving the fragment a

‘disjunct’ quality (see again Supko, 2017). In this clip, besides a large use of the chromatic (and also whole-tone) scale, one can also note several tonal elements, such as for example a diminished harmony and resolving notes in the last measure of the fragment (measure 41). Also, the interplay between the melodic trajectories of the two hands is characteristic. We agree with Supko that there is a strong sense of inevitability within this fragment, and that however “it seems clear that the passage’s inevitability derives more from the consistent intervallic and rhythmic patterning than from any sense of anticipation arising from the composite harmony.” (Supko, 2017, p. 24). The lack of a clear direction, in both harmonic and dynamic-rhythmic terms, could underlie the scarcity of polarized ratings in the OD.

Notable associations/repulsions: Although the dimensions are not very polarized in the OD, the clip resulted close to several of the paintings when considering the Euclidean distance: above all, *Black spot* (1) and *Picture with a white border* (5).

Comparison with Direct Association results, and pictorial analysis of the associated paintings: In *Picture with a white border* a spear and a trumpet are emphasized; there is a contrast between the white color and the dark background; Kandinsky used patches of unrelated color- white, pale blue, and pink- to create a feeling of transparency in most of the trumpet's area. In *Black spot* Kandinsky definitively abandoned the figurative elements, of which remain only a few hints, recognizable in the lower left (houses, trees), while the black color conveys mourning and anguish. Both paintings were difficult for the subjects to rate with one of the extremes of each dimension, which could be the main reason for the association with the clip in terms of Euclidean distance, which, however, was not mirrored by any trend in the results of Task 2.

03. *Mäßig* (bars 53-58)

Osgood Differential results: With the Osgood semantic differential this clip was associated only with the adjective cupo (gloomy). The ratings of grave and angoscioso (distressing), as hypothesized, were also very close to statistical threshold.

Musical analysis: In this fragment we hear another repetition of the main theme followed by a dramatic crescendo, with octaves in the left hand, alternating with tonal-reminiscent chords (although not in a tonal succession) played by the right hand, that culminates in a brief ostinato played by the right hand in *marcato* articulation. It is somehow surprising (differently than for clip no. 02) that also this fragment did not receive a large number of significant ratings with respect to the tested dimensions, because of the clearly directional *crescendo* that it includes. However, considering precisely the heterogeneous nature of the clip given by the contrast between the first two measures and the following, it is possible that subjects, trying to consider the local features of the clip instead of the global behavior of the fragment, rated it as ambiguous. In particular, the major seventh-like chord appearing at the end of measure 54 heavily contrasts with the minor-sounding harmony of the upcoming measures.

Comparison with Direct Association results, and notable associations/repulsions: In Task 2 this clip was positively associated in a highly significant manner with *Lake boat trip* (the residual was 3.08). Interestingly, *Lake boat trip* was also the painting with the lowest distance, confirming the direct association. Therefore, in this case, the association seems to be mediated by the dimensions included in the OD, although they were not markedly polarized in the ratings. Rather, it is precisely the ambiguous/heterogeneous nature of both the clip and the painting that could have mediated the association. The clip was also significantly associated with *Composition 6* (2.50), as well as significantly repelled by *Black spot* (-2.37). As to the painting *Black spot,* the color black (for Kandinsky conveying anguish and mourning) predominates, and the spot is placed in a central position.

04. *Sehr langsam* (bars 2-5)

Osgood Differential results: With the Osgood semantic differential, this clip was rated as spento (faded), calmo (calm), grave, cupo (gloomy), morbido (soft), piacevole (pleasant), and statico (static). The rating of consonante was very close to threshold level.

Musical analysis: The adjectives are very well suited to the character of the fragment, and the rating of morbido is especially interesting to be related, possibly, to the slow ostinato motif played by the left hand in the low register throughout most of the clip. The fragment consists of part of the introduction of the second piece (*sehr langsam*). At measure 4, the main theme of the first piece is reprised in a modified version, briefly interrupting the ostinato. The clip is characterized by its very slow tempo (*sehr langsam*, *mäßig*) and, again, the long ostinato. Also, here tonal elements such as note resolutions are evident in the writing.

Comparison with Direct Association, notable associations/repulsions, and pictorial analysis of the associated paintings: The clip was consistently repelled by *Composition 8* in the results of both tasks. The bright colors of the painting, and especially the background (which was rated with adjectives such as acceso (fiery), luminoso (bright), piacevole (pleasant)), are in contrast with the cupo (gloomy) rating of the clip, and, cross-modally, with the rating of grave; when considering the 14 clips, the grave-acuto couple is highly correlated with the cupo-luminoso (gloomy-bright) couple ($r = 0.95$) (see also, for example, the results of Albertazzi et al., 2016a). The dynamicity of the shapes on the painting and, even more, the sharp angles and lines is also in contrast with the rating of calmo (calm) that follows the slow, repetitive, and quiet character of the musical fragment. Furthermore, the strong geometrical character of *Composition 8* seems to contrast with the relatively flexible agogics of this clip, especially when directly matched (see below comments about *Musette*, and *Composition 8*).

In the results of Task 2, the clip was also positively associated with two paintings: *Lake boat trip* (15) and *Improvisation 19* (11). In *Lake boat trip*, the light filtering from the center of the canvas, on a dark background, gives the impression of a shady landscape, perhaps a sunset, which well reflects the ratings obtained with the OD. The same holds for *Improvisation 19*, in which the crepuscular background colors are covered by black vertical (and therefore steady) figures. As to the basic Kandinsky contraries of the contradictions (1913), in *Lake boat trip* the masses, the thickness (amorphous patches of color), the color, the coldness and darkness (conveyed by the blacks and the dark blues) prevail. These visual characteristics of the painting crossmodally map the acoustic patterns.

Interestingly, the Euclidean distance did not reflect the strong associations with both paintings, and in particular with *Lake boat trip*. This would suggest that the stimuli, when rated alone, did not exhibit the same features of their concurrent presentation (or also that, less likely, the OD dimensions were not enough to capture them). For example, the case of *Lake boat trip* and *Improvisation 19* are interesting because they did not exhibit very polarized ratings with respect to the OD, perhaps due to the possibility for an open interpretation of the figurative elements in the painting. As regards *Improvisation 19*, the warmer colors could have also contributed to opposite ratings in the OD, despite the strong direct matching.

05. *Sehr langsam* (bars 10-13)

Osgood Differential results: With the Osgood semantic differential, surprisingly, this clip did not exceed statistical threshold with respect to any of the ratings. Although no rating was statistically significant, the adjectives cupo and angoscioso seem to show a moderate attraction for the clip.

Musical analysis: In this excerpt, the theme is first reprised in a new variation. After a small crescendo, a sudden outbreak of *marcato* chords creates a moment of tension. The chords have a particularly tonal character which drives the succession until the last one, which resounds like a dramatic minor ninth chord (although the major sixth in the bass contributes in ebbing the atmosphere). The register is mixed between both hands and the timbre rich.

Notable associations/repulsions and comparison with Direct Association results: This clip was semantically similar to *Lake boat trip* (15) with respect to the Euclidean distance, although some of the pairs showing the most similar ratings were again in the "neutral" band sereno/angoscioso (serene/distressing), caldo/freddo (warm/cold), consonante-dissonante (consonant/dissonant), among the polarized ratings, probably reflecting disagreement among subjects (see Results). The stimuli were both rated as grave, cupo (gloomy), duro (hard), and statico (static). Coherently with this result, the couple clip 9 and *Lake boat trip* were also significantly associated in Task 2 (see below and Supplemental Table 2).

06. *Sehr langsam* (bars 43-47)

Osgood Differential results: With the Osgood semantic differential, this clip was rated as acceso (fiery), agitato (agitated), acuto, luminoso (bright), piacevole (pleasant), and dinamico (dynamic).

Musical analysis: This is indeed a very salient excerpt. It starts with a crescendo and a chromatic ascension of the melodic pattern in both hands. Then it culminates in a series of trills accompanied by an ostinato motif in the left hand, containing arpeggiated *fortissimo* chords. It eventually fades into another chromatic run, *decrescendo*, played by the right hand only.

The rating of acceso (fiery) has been most likely driven by the fast trills as well as the overall fluid, although dramatic, character of the melody. Indeed, the fragment was perceived as dynamic,

which can be explained by the crescendo, extreme dynamics, combined with the incessantly rising chromatisms. The overall tense character of the fragment seems to be also partially reflected in the agitato (agitated) rating. As to the piacevole (pleasant) rating, the very peculiar ornament of the trill may have contributed to eliciting "pleasure" feelings in the subjects, or at least their better enjoyment of the clip.

Notable associations/repulsions and comparison with Direct Association results: The fragment was semantically similar to *Improvisation (Gorge)* considering the Euclidean distance, although in the results of Task 2 *Improvisation (Gorge)* was only the second-most attracted painting among the three alternatives. Among the common associations that were polarized to one of the extremes are acceso (fiery), agitato (agitated), piacevole (pleasant), and dinamico (dynamic). Stylistic elements that may be responsible for such ratings are the vivid colors in the painting as well as the richness and variety of dynamic shapes that spread through the painting.

07. *Bewegt* (bars 1-5)

Osgood Differential results: With the Osgood semantic differential, this clip received several statistically significant ratings, as acceso (fiery), agitato (agitated), dissonante (dissonant), aspro (bitter) duro (hard), angoscioso (distressing), and dinamico (dynamic).

Musical analysis: The fragment contains the first five measures of the third piece (*bewegt*). According to Haimo (1990), this is the only one of the three pieces that does not show any thematic material. It is dynamically extreme and, because of the rhythmic conflict between overlapping lines (which goes well with ratings of aspro (bitter), duro (hard), and perhaps dissonante (dissonant)) the main meter is very difficult to discern. The register is very extended and contributes to creating a very full and dense timbre.

Notable associations/repulsions: According to the Euclidean distance, the clip was semantically different with respect to several paintings, among which *Sketch for Composition 2* and *Landscape with red spots*. Among the polarized dimensions, the repulsion was mostly mediated by ratings such as acceso (fiery) and luminoso (bright) for the two paintings, and an overall polarization towards the "positive" extreme of the couples for the painting, which is opposite to what happened with the clip.

Comparison with Direct Association results: As to the results of Task 2, the painting *Sketch for Composition 2* was also repelled by the clip, although not significantly. A much more moderate effect was found for *Landscape with red spots*. A weaker repulsion between the latter and the clip could have been caused by the direct interaction of figural elements, such as the horse and the moving human-shaped figures, with highly iconic musical elements such as the ternary figures in the first part of the clip.

08. *Bewegt* (bars 32-35)

Osgood Differential results: With the Osgood semantic differential, this clip was rated as grave, cupo (gloomy), and angoscioso (distressing).

Musical analysis: An ostinato motif starts suddenly very loud, decreasing in intensity repetition after repetition. It consists of three notes, a melodic tritone-fourth played descending by the right hand in middle register, and ascending in low register by the left hand, giving an overall low tone to the fragment (which goes together with the rating of grave and cupo). The last repetition, as a bottom line, happens at half speed and staccato articulation, with extremely soft dynamics (*pppp*). The ostinato is therefore very prolonged and explicit, further increasing the tension. After a pause, a few closing notes, both high and low, are played by the alternating hands with very soft dynamics.

Comparison with Direct Association results and notable associations/repulsions: The clip was significantly attracted by *Impression 3 (Concert*) in the results of the direct association (Task 2), and at the same time repelled in the results of the OD (Task 1). This interesting result may be triggered by a *Gestalt* effect resulting from the presentation of the painting together with the music, that acquires qualities not existing in the stimuli in isolation. For example, the color yellow can be perceived as fiery, warm, and bright (as well as "happy"), or dramatic and scary, depending on the perceptual context, which in this case may be cross-modally determined by the agitated musical fragment. Last but not least, the participants, naïve as to Kandinsky's paintings, may have been led to their evaluation by the salient presence of the black mass.
As in the case of clip 07, also taken from the same movement, this fragment was numerically (but not significantly) repelled by *Sketch for Composition 2*, which is again explainable in terms of a general inversion in the polarization of the couples, and especially luminoso/cupo (bright/gloomy). If on the clip side the cupo (gloomy) adjective can be mostly related to the low register of the main pattern in the fragment (see previous comments), the notably strong luminoso (bright) rating of the painting could be related to the use of very saturated colors in the painting.

***Suite for Piano*, Op.25:**

09. *Gavotte* (bars 8-16)

Osgood Differential results: With the Osgood semantic differential, the clip was rated as acceso (fiery), piacevole (pleasant), and dinamico (dynamic).

Musical analysis: The fragment, taken from the second movement of the *Suite*, corresponds to the second section. Here the rhythm closely resembles the Baroque dance of which the

movement carries the name, based on dactylic figures. The motif is mainly based on four notes (one of the sub-groups of the basic series of the Suite; for more details, see Mayhew, 1962) that are repeated as an ostinato by the left hand, while the right hand moves in a higher register. A partial climax created by the raising register happens at measure 12, followed by decrease in register, dynamics and tempo (*p*, *dolce*). Rhythmic figures, involving the interplay between different voices, are very explicit and "catchy", which could explain the rating of piacevole, and also acceso (fiery) and dinamico (dynamic) despite the overall light dynamics.

Notable associations/repulsions: The Gavotte fragment was semantically closest to *Composition 5*, when considering the Euclidean distance. Among those polarized on one side, dimensions that accounted for the association are acceso (fiery), dinamico (dynamic), and piacevole (pleasant).

Pictorial analysis of the associated paintings: The rating of acceso (fiery) is surprising with respect to the tenuous (although bright) unsaturated colors, however it might have been influenced by the free diagonal movement of the black crisscrossing lines on the canvas and specifically by the thick curving black line extending from upper right and moving to left center (which in turn gives the painting its dynamic character), and by the swirling mass of amorphous patches of color. However, as regards the piacevole/spiacevole (pleasant/unpleasant) couple, as mentioned earlier, it is possible that subjects expressed their personal enjoyment of the stimuli, especially as regards the painting.

Comparison with Direct Association results: However, these results do not correspond to those obtained by the direct association in Task 2, which highlighted a repulsion with *Improvisation 19* and a positive association with *Composition 8.*

10. *Musette* (bars 1-9)

Osgood Differential results: With the Osgood semantic differential, this clip was rated as acceso (fiery), agitato (agitated), aspro (bitter), acuto, and dinamico (dynamic).

Musical analysis: The clip is extracted from the third movement of the *Suite*, another binary dance, with a character similar to that of the *Gavotte*, which could again explain the acceso (fiery), agitato (agitated), and dinamico (dynamic) rating. The melodic lines, as in the case of the previous fragments, are mostly based on the same thematic material (third group of the basic series). The timbre is thin due to the high register in which both hands are playing, which could have likely led to the rating of acuto, and the *staccato* articulation. The same features could have also driven the rating of aspro (bitter).

Comparison with Direct Association results, and notable associations:

In Task 2, the clip was directly associated with *First abstract watercolor, Improvisation (Gorge),* and to a lesser extent with *Composition 8*. As to the Euclidean distance, the situation is less clear, the *Musette* being moderately close to several of the paintings (among which, *First abstract watercolor*, and, to a lesser extent, *Composition 8*). The fact that two associations were strong in Task 2 and not in Task 1 (by means of the Euclidean distance) may be due to the fact that the dimensions used to rate the stimuli in the OD were not enough to capture the similarity between the stimuli highlighted by direct association; or that the stimuli presented in combinations could create configurations not found in the stimuli in isolation (see general comments later). For example, while listening to the *Musette*, the two paintings (and especially *Composition 8*) seem to come alive resounding in their own motion.

Pictorial analysis of the associated paintings: The presence of more geometrical elements in *Composition 8* makes its movement more difficult to appreciate when looking at the painting alone (because somehow less “ecological”). However, precisely its geometrical but dynamic character emerges when matched to the *Musette*, also stressed by the strict tempo, the

staccato articulation, and the high register (associated with sharp/acute angles; in this case, "acute" was also a rating in the OD for both stimuli).

As to *First abstract watercolor*, the individuation of the constitutive elements of shape, devoid of any trait of objectivity, is clearly retrievable. This painting, in fact, shows a dynamic and agitated character, although, besides lines, masses and strokes are still present as configurational elements. As to the association of the clip with *Improvisation (Gorge)*, the representation of a chaotic environment in the painting is well mapped by its rating in Task 1 as acceso (fiery), conveyed by the red and yellows and bright blues, dinamico (dynamic) and agitato (agitated). In this painting, as for *First abstract watercolor*, masses are still present in the forms of colored areas, but the linear elements progressively gain ground. *Composition 8,* then, is characterized by figural linear forms such as circles (in particular) or triangles, and by the interplay of straight and curvilinear lines. The architectural composition of this painting (that shows the progressive achievement by Kandinsky of the basic elements of shape as a product of a reflection on the content of the impressions and the improvisations of other paintings) is surely dynamic in the interplay between round and angular forms, tips, traits and circles/semicircles; and acute in the essential reduction of shape to thin linear elements. Masses (colored areas), in fact, with the main exclusion of the circles on top left of the painting, are not dominant.

11. *Intermezzo* (bars 1-3)

Osgood Differential results: With the Osgood semantic differential, the clip was not surprisingly rated as spento (faded), calmo (calm), morbido (soft), piacevole (pleasant), and statico (static).

Musical analysis: The excerpt pertains to the slow *Intermezzo*, of romantic reminiscence, employing a structure freer than the Baroque dances in the *Suite*. The fragment comprises the first three measures, in which the basic series appears arranged in an ingenious way: the first

notes of the series appear in a repeating pedal figure, below which the main theme of the movement, made of the following notes of the series, is played by the left hand. The articulation is soft and smooth (*pp*, *espressivo*).

Comparison with Direct Association results, and notable associations: The *Intermezzo* was significantly repelled by *Improvisation (Gorge*) in the direct association of Task 2. The same trend happens by means of the Euclidean distance, showing a high distance also with *Composition 8* (also mirrored by the Euclidean distance). These strong repulsions clearly result from the opposition between the overall positive and passive character of the clip with the dense, dynamic, and colored content of the paintings. Furthermore, in both tasks the painting *Improvisation 14* showed the highest degree of attraction with the clip with respect to the alternative paintings. The attraction could have been partly induced by the unsaturated colors matching with the slow and quiet atmosphere of the musical fragment.

12. *Menuett* (bars 1-7)

Osgood Differential results: With the Osgood semantic differential, this clip was associated with the adjectives dolce (sweet), piacevole (pleasant), and sereno (serene).

Musical analysis: The fragment consists of the first measures of the *Minuetto*, where the ternary meter of the dance is very evident. The melody is articulated in a delicate and intimate way (*p*, *innig*), while the parts are arranged to provide timbral variations and widely spaced between each other (see Mayhew, 1962). The right hand plays a contrapuntal game by splitting the sub-groups of the series into different voices, while the left-hand reprises and elaborates another sub-group of the series. Therefore, the melodic and timbral richness of the fragment could have possibly resulted in an overall positive evaluation by the subjects.

Comparison with Direct Association results, and notable associations: There was no noticeable closeness between the clip and the paintings, although the two closest were *Improvisation 14* and *Improvisation 26 (Rowing)*, perhaps explained by a general moderate polarization towards a "pleasant" extreme (e.g. soft, pleasant, serene, although not necessarily statistically significant). In Task 2, there were two significant attractions with *Landscape with red spots*, 1912 (2.11) and with *Improvisation 10* (2.07), both characterized by bright and sunny colors and landscape elements. To be noted is that, according to Kandinsky, the color red is warm, expands and advances in pictorial space towards the viewer (while the color blue is cold, shrinks and retreats). Also, in this case a significant repulsion between the clip and the painting *Improvisation (Gorge)* (the standardized residual was -2.51), confirmed by the Euclidean distance, can be identified. The repulsion could have been mediated by the elegant and simple rhythm of the *Minuet*, which contrasts with the crowded and chaotic character of the painting.

13. *Trio* (bars 34-42)

Osgood Differential results: With the Osgood semantic differential, the clip, the beginning of the *Trio* inside the *Minuetto*, was rated as acceso (fiery), agitato (agitated), duro (hard), and dinamico (dynamic), with also a slight displacement towards the adjectives dissonante (dissonant), aspro (bitter), and acuto (not exceeding threshold level).

Musical analysis: The ratings are understandable since, differently from the previous fragment, this one presents very sharp articulation (*f*, *martellato*). The melody, a mirror canon built over the basic series and distributed on both hands in different registers, is arranged in leaps, so that the main lines sound broken.

Notable associations/repulsions and comparison with Direct Association results: Oppositely to the previous clip, extracted from the *Minuet*, this fragment was the closest to *Improvisation*

(Gorge) as regards Euclidean distance. The association well mirrors the opposite character of this clip with respect to the previous one and is well understandable in terms of the stylistic features of *Improvisation (Gorge)* (see above). The results of Task 2 confirm the attraction between clip 13 and *Improvisation (Gorge)* with a statistically significant positive residual (2.65).

14. *Gigue* (bars 1-13)

Osgood Differential results: With the Osgood semantic differential, the clip was rated as acceso (fiery), agitato (agitated), dissonante (dissonant), duro (hard), and dinamico (dynamic).

Musical analysis: The fragment corresponds to the first measures of the *Gig*a, the most agitated of the pieces (*rasch*), and the conclusive movement of the suite. Differently from the other movements, the rhythmic structure is quite different from its Baroque counterpart. Although ternary figures appear sporadically, the main meter is binary and there is much rhythmic variation during the piece. Overall, it appears as much more disordered than the other movements. In the first measures, comprised in the clip, the notes of the basic series appear in a disjunct formation (see Mayhew, 1962) that renders the theme not recognizable. Until measure 10 the dynamic is strong (*ff*), decreasing from measure 10 (*pp* and *poco ritardando*). However, the abrupt dynamic change, rather than softening the atmosphere, ends up by increasing the tension further. The adjectives chosen by the subjects in Task 1 seem therefore to match the characteristics of the fragment well.

Comparison with Direct Association results, and notable associations/repulsions: Not surprisingly, the painting *Improvisation (Gorge)* was highly significantly associated also with this clip in Task 2 (4.30). This clip was also associated, although to a lesser extent, with *First abstract watercolor*, the painting showing an overall dynamic structure, although less extreme and with overall darker color content. Both paintings were the ones showing the smallest Euclidean

distance from the clip. The clip resulted significantly repelled in Task 2 from the painting *Lake boat trip* and from the painting *Improvisation 19*. This last painting was one of the most distant from clip 14 (perhaps because of its vertical, static lines and figures, and relatively uniform color content), while this was not true for *Lake boat trip*. It is possible that for this painting the repulsion was mediated by dimensions not included in the chosen list of OD or by the failed attempt to directly match figural elements such as the boats on the flat water, with the turbulent character of the clip.

General comment on "central" ratings:

On looking at the standard deviation of the ratings in relation to their mean (Supplemental Table 1-2), it is possible to observe that, on average, the variability is higher for ratings in the center of the range (e.g. neither hot nor cold). This reflects a disagreement of the subjects with respect to the dimensions that resulted not polarized to one of the two sides, and therefore likely to be more influenced by top-down factors than perceptual ones. As a consequence of this observation, in commenting on the associations mediated by the OD we gave the priority to those dimensions where the ratings were polarized to one of the two extremes.
